# MAP7 family proteins regulate kinesin-1 recruitment and activation

**DOI:** 10.1083/jcb.201808065

**Published:** 2019-02-15

**Authors:** Peter Jan Hooikaas, Maud Martin, Tobias Mühlethaler, Gert-Jan Kuijntjes, Cathelijn A.E. Peeters, Eugene A. Katrukha, Luca Ferrari, Riccardo Stucchi, Daan G.F. Verhagen, Wilhelmina E. van Riel, Ilya Grigoriev, A.F. Maarten Altelaar, Casper C. Hoogenraad, Stefan G.D. Rüdiger, Michel O. Steinmetz, Lukas C. Kapitein, Anna Akhmanova

**Affiliations:** 1 Cell Biology, Department of Biology, Faculty of Science, Utrecht University, Utrecht, Netherlands; 2 Laboratory of Biomolecular Research, Division of Biology and Chemistry, Paul Scherrer Institut, Villigen, Switzerland; 3 Cellular Protein Chemistry, Bijvoet Center for Biomolecular Research, Utrecht University, Utrecht, Netherlands; 4 Biomolecular Mass Spectrometry and Proteomics, Bijvoet Center for Biomolecular Research, Utrecht Institute for Pharmaceutical Sciences and The Netherlands Proteomics Centre, Utrecht University, Utrecht, Netherlands; 5 Biozentrum, University of Basel, Basel, Switzerland

## Abstract

Kinesin-1 is responsible for microtubule-based transport of numerous cellular cargoes. Here, we explored the regulation of kinesin-1 by MAP7 proteins. We found that all four mammalian MAP7 family members bind to kinesin-1. In HeLa cells, MAP7, MAP7D1, and MAP7D3 act redundantly to enable kinesin-1–dependent transport and microtubule recruitment of the truncated kinesin-1 KIF5B-560, which contains the stalk but not the cargo-binding and autoregulatory regions. In vitro, purified MAP7 and MAP7D3 increase microtubule landing rate and processivity of kinesin-1 through transient association with the motor. MAP7 proteins promote binding of kinesin-1 to microtubules both directly, through the N-terminal microtubule-binding domain and unstructured linker region, and indirectly, through an allosteric effect exerted by the kinesin-binding C-terminal domain. Compared with MAP7, MAP7D3 has a higher affinity for kinesin-1 and a lower affinity for microtubules and, unlike MAP7, can be cotransported with the motor. We propose that MAP7 proteins are microtubule-tethered kinesin-1 activators, with which the motor transiently interacts as it moves along microtubules.

## Abstract (precis)

Hooikaas et al. show that mammalian MAP7 family proteins act redundantly to activate the kinesin-1 motor protein. Using experiments in cells and in vitro reconstitution assays, they demonstrate that MAP7 proteins promote microtubule recruitment and processivity of kinesin-1 by transiently associating with the stalk region of the motor.

## Introduction

Kinesins are molecular motors responsible for the transport of different organelles and macromolecular complexes along microtubules (MTs) and for controlling MT organization and dynamics (Verhey et al., 2011; Hirokawa and Tanaka, 2015). The spatial and temporal control of kinesin localization and activity depends on numerous factors, such as cargo adaptors, posttranslational modifications, and the interactions with MT-associated proteins (MAPs; Verhey and Hammond, 2009; Akhmanova and Hammer, 2010; Fu and Holzbaur, 2014; Barlan and Gelfand, 2017).

**Figure 1.**
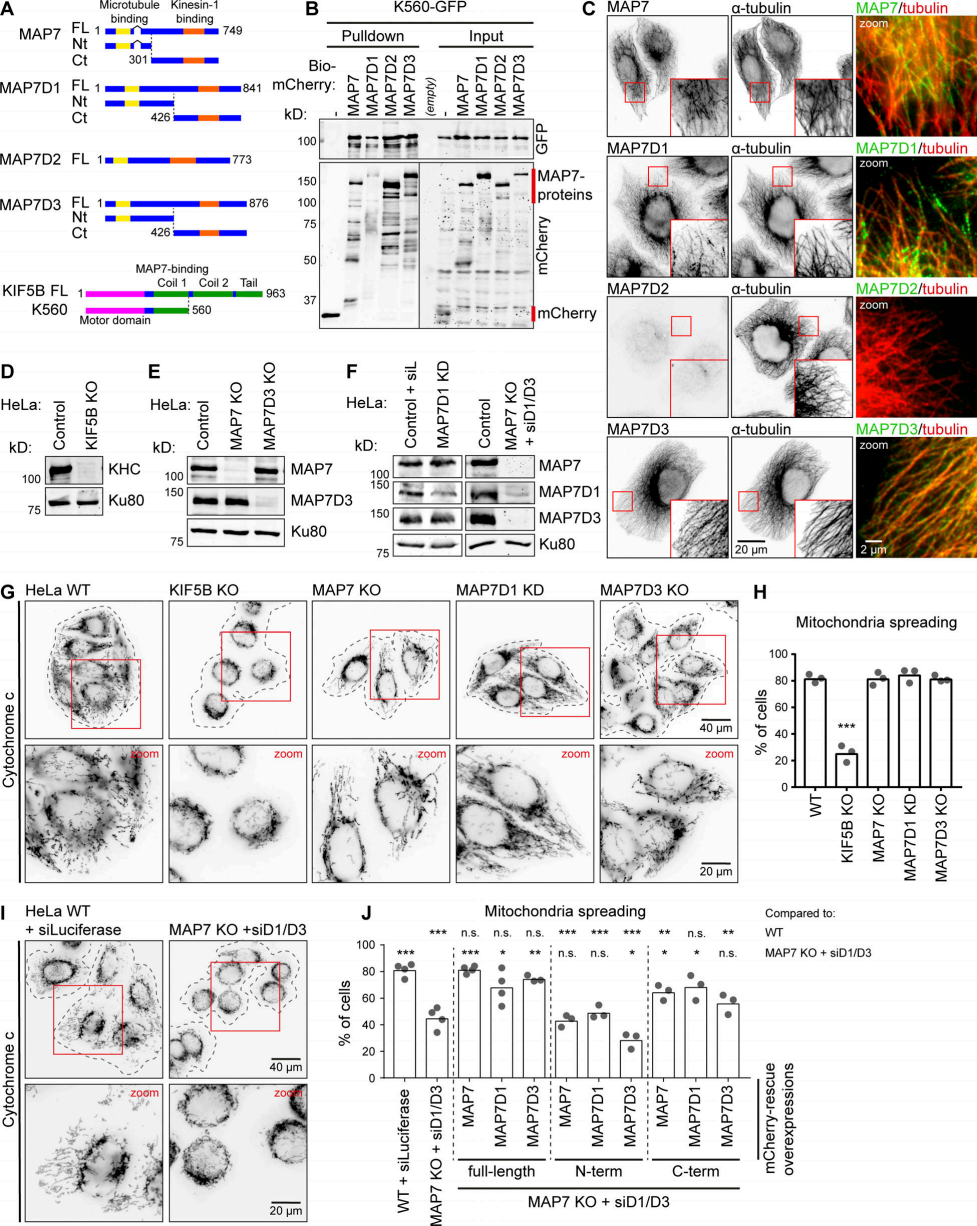
**Redundant function of MAP7 family proteins in kinesin-1–dependent mitochondria distribution. (A)** Schemes of MAP7 family proteins and KIF5B constructs. **(B)** Streptavidin pull-down assay with extracts of HEK293T cells expressing BirA, K560-GFP (prey), and the indicated Bio-mCherry–labeled proteins (bait) analyzed by Western blotting. Red lines indicate the position of mCherry (negative control) and MAP7 proteins. **(C)** Immunostaining of HeLa cells for endogenous MAP7 family members and α-tubulin imaged on a widefield microscope. **(D–F)** Western blot analysis of the indicated HeLa knockout (KO) and knockdown (KD) cells with the indicated antibodies; Ku80 was used as a loading control. **(G and I)** HeLa cells treated as indicated stained for mitochondria (cytochrome *c*). Cell outlines are indicated with gray dashed lines; zooms (red squares) are shown below. **(H and J)** Mitochondria distribution scored per condition using cytochrome *c* staining. (H) *n* = 345, 347, 332, 338, and 373 cells from three independent experiments; WT vs KIF5B KO, P = 0.0002, *t* test. (J) *n* = 444 (WT + siLuciferase), *n* = 589 (MAP7 KO + siMAP7D1/D3), and for rescue conditions on top of MAP7 KO + siMAP7D1/D3: *n* = 261, 277, 324, 297, 277, 296, 263, 267, and 416 cells all from three or four independent experiments. *, P < 0.05; **, P < 0.01; ***, P < 0.001; *t* test. Ct, C terminal; FL, full length; Nt, N terminus.

Kinesin-1 is the major MT plus-end–directed motor involved in a broad variety of transport processes (Akhmanova and Hammer, 2010; Verhey et al., 2011; Hirokawa and Tanaka, 2015). This motor is well known to be regulated by different MAPs. The neuronal MAPs tau and MAP2 inhibit kinesin-1–driven motility (Ebneth et al., 1998; Trinczek et al., 1999; Seitz et al., 2002; Vershinin et al., 2007; Dixit et al., 2008; Gumy et al., 2017; Monroy et al., 2018). In contrast, MAP7 family members are firmly established to be positive regulators of kinesin-1 (Sung et al., 2008; Metzger et al., 2012; Barlan et al., 2013; Metivier et al., 2018; Monroy et al., 2018). MAP7 proteins are represented by a single homologue (ensconsin) in flies and by four isoforms encoded by different genes (MAP7, MAP7D1, MAP7D2, and MAP7D3) in mammals (Bulinski and Bossler, 1994; Metzger et al., 2012; Yadav et al., 2014). All MAP7 family members have a similar organization, with two conserved domains that are predicted to be helical, connected by an unstructured linker. The N-terminal domain of MAP7 proteins strongly interacts with MTs, while the C-terminal domain binds to the stalk region of kinesin-1 (Sun et al., 2011; Metzger et al., 2012; Monroy et al., 2018; Fig. 1 A). Additional regions with MT affinity were found in the linker of MAP7 and the C-terminal part of MAP7D3 (Yadav et al., 2014; Tymanskyj et al., 2018). In flies, ensconsin is an essential kinesin-1 cofactor required for numerous processes ranging from organelle transport to MT sliding (Sung et al., 2008; Metzger et al., 2012; Barlan et al., 2013; Metivier et al., 2018; Monroy et al., 2018). In mammalian myotubes, MAP7 is needed for proper kinesin-1–dependent nuclear distribution (Metzger et al., 2012), but whether MAP7 proteins are needed for other kinesin-1–dependent processes in mammals has not been investigated. It is also unknown whether mammalian MAP7 homologues all behave similarly and whether they have different, overlapping or redundant functions.

Interestingly, in vitro experiments in fly ovary extracts have shown that the full-length kinesin-1, but not its minimal dimeric kinesin-1 fragment, requires ensconsin for productive interaction with MTs (Sung et al., 2008). In vitro reconstitutions with purified proteins demonstrated that MAP7 recruited kinesin-1 to MTs and somewhat decreased motor velocity but had only a mild effect on kinesin-1 run length (Monroy et al., 2018). Importantly, MAP7 was highly immobile in these assays and was not cotransported with the motor, suggesting that MAP7 affects the initial recruitment of the kinesin to MTs but has little impact on kinesin-1 movement (Monroy et al., 2018). However, some observations in flies do not agree with this simple model, as it was shown that the C-terminal fragment of ensconsin, which misses the MT-binding domain (Sung et al., 2008), significantly rescues kinesin-1–related transport deficiencies in cells lacking ensconsin (Barlan et al., 2013; Metivier et al., 2018). Kinesin-1 is well known to be autoinhibited by its C-terminal cargo-binding domains (Verhey and Hammond, 2009), and it was proposed that ensconsin plays a role in relieving autoinhibition of the kinesin (Barlan et al., 2013). This possibility is in line with the experiments performed in extracts (Sung et al., 2008) but was not yet tested with purified proteins.

Here, we explored the relationship between kinesin-1 activity and mammalian MAP7 proteins. We found that MAP7 family members act redundantly to promote kinesin-1–dependent distribution of mitochondria, as well as MT binding of kinesin-1 KIF5B fragment 1–560 (K560; Case et al., 1997), which contains the motor domain and the dimerizing stalk with the MAP7-binding site, but not the cargo-binding and autoinhibitory domains. MT recruitment of K560 was rescued not only by full-length MAP7s but also by their C-terminal domains, which lacked the major MT-binding region. These results were recapitulated using in vitro reconstitution assays with purified proteins, which provided evidence both for MT tethering and allosteric activation of kinesin-1 by MAP7 family members. In agreement with published data, we found that MAP7 was immobile on MTs in vitro (Monroy et al., 2018), whereas MAP7D3 could be observed moving together with K560 motors. In spite of these differences, both MAPs increased not only the recruitment of kinesin-1 to MTs but also its processivity. Such an effect can be explained if the interaction between MAP7 and kinesin-1 is weak and transient, and this was confirmed by biochemical and imaging experiments. Taken together, our data show that MAP7 proteins redundantly regulate kinesin-1-dependent transport by acting as MT-tethered recruitment factors and activators of this kinesin.

## Results

### MAP7 family members act redundantly in mitochondrial distribution in HeLa cells

To test whether all four MAP7 family members can potentially act as kinesin-1 regulators, we performed a pull-down assay and found that all four MAP7 proteins could bind to the kinesin-1 deletion mutant K560 (Fig. 1, A and B). Gene expression analysis at the mRNA and protein level indicated that HeLa cells coexpress MAP7, MAP7D1, and MAP7D3 (Syred et al., 2013; Kikuchi et al., 2018), and we confirmed these data by antibody staining (Fig. 1 C). In contrast, MAP7D2, which is highly expressed in brain tissue (Niida and Yachie, 2011), was not expressed in HeLa cells. To test if all three MAP7s are required for kinesin-1 function, we initially used the distribution of mitochondria as readout, because it strongly depends on kinesin-1 KIF5B (Tanaka et al., 1998). In the absence of KIF5B, mitochondria were no longer dispersed in the cytoplasm but were clustered around the nucleus (Fig. 1, D, G, and H). Next, we generated HeLa cells lacking each individual MAP7 family member. In these cells, the expression of the remaining MAP7s was not altered, and no defects in the localization of mitochondria were observed (Fig. 1, E–H).

We next attempted to generate a stable triple knockout of MAP7, MAP7D1, and MAP7D3, but such cells were not viable. It is unlikely that this was due to the lack of kinesin-1–mediated transport, as KIF5B knockout cells displayed no apparent growth or proliferation defects, and the two other kinesin-1 isoforms, KIF5A and KIF5C, do not seem to be expressed in HeLa cells (Nagaraj et al., 2011). Although MAP7 was shown to be phosphorylated and thus inactivated during mitosis (Mchedlishvili et al., 2018), it is possible that MAP7 proteins still contribute to cell division, as ensconsin is known to participate in spindle formation in flies (Gallaud et al., 2014), and MAP7D3 was reported to modulate the recruitment of kinesin-13 to the mitotic spindle (Kwon et al., 2016). To remove all three MAP7 homologues simultaneously, we performed siRNA-mediated knockdown of MAP7D1 and MAP7D3 in the stable MAP7 knockout line, and this approach resulted in an efficient loss of all three MAP7 family members (Fig. 1 F). Depletion of all three MAP7 homologues mimicked the effect of KIF5B knockout, leading to a strong perinuclear clustering of mitochondria (Fig. 1, I and J). To exclude that this phenotype was caused by defects in MT organization, we assessed it by antibody staining and found that the overall MT arrangement and density were similar (Fig. S1, A and B). Furthermore, live imaging of EB3-GFP showed no differences in MT plus-end growth, and polymerizing MT ends still reached the cell periphery (Fig. S1, C–E). We conclude that MAP7 family members act redundantly in mitochondria localization and that this effect is unlikely to be due to alterations in MT network architecture.

Mitochondrial positioning in these cells was rescued by reexpressing individual full length MAP7 proteins, and it was also partially rescued by expressing the C termini of MAP7 and MAP7D1 (Fig. 1 J). Rescue with the MAP7D3–C terminus (Ct) was less efficient, because the construct was mostly accumulated in the nucleus, and, as its concentration in the cytoplasm was low, only highly expressing cells showed rescue (Fig. S1 F).

**Figure 2.**
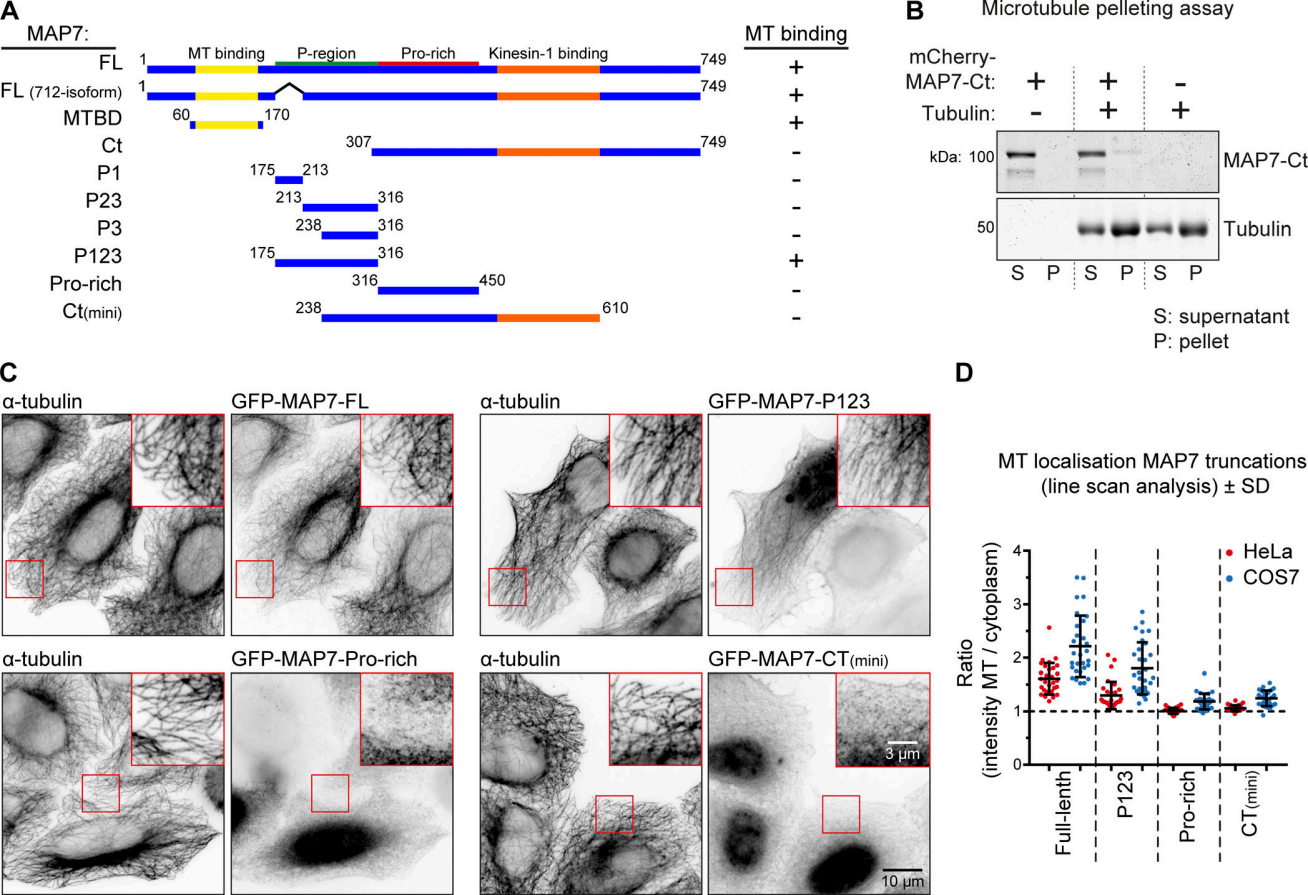
**Characterization of MT-binding domains of MAP7. (A)** Scheme of MAP7 truncations. MT binding was assayed by overexpression of GFP-tagged constructs and costain for α-tubulin; examples are shown in C. FL, full length; MTBD, MT binding domain. **(B)** MT pelleting assay with mCherry-MAP7-Ct analyzed by SDS-PAGE. Uncropped gel images are shown in Fig. S1 G. **(C and D)** Indicated GFP-tagged MAP7 constructs were overexpressed in MAP7 knockout HeLa cells costained for α-tubulin (C) to quantify their MT enrichment by line-scan analysis (D). *n* = 10 cells representing 30 MTs (3 per cell) per condition.

Rescue of kinesin-1 function by MAP7-Ct could be potentially explained by their residual affinity for MTs. For example, recent work has demonstrated that MAP7 contains an additional MT binding domain (termed “P-region”) within the intrinsically disordered linker part of the protein (Tymanskyj et al., 2018; Fig. 2 A). To address this possibility, we systematically examined the ability of different parts of the MAP7 linker to interact with MTs (Fig. 2 A). Using a MT pelleting assay, we found that the MAP7-Ct fragment used in cellular experiments (Fig. 1, A and J) did not cosediment with MTs (Figs. 2 B and S1 G). We were also unable to detect the binding of the mCherry-tagged version of this fragment to MTs in vitro (Fig. S1 H). Next, we tested MT binding of different MAP7 fragments by overexpression of their GFP-tagged fusions. Although we could reconfirm MT affinity of the P-region, its smaller fragments as well as other MAP7 deletion mutants lacking the N-terminal MT binding domain, including the MAP7-Ct and MAP7-Ct(mini), showed no MT enrichment in HeLa cells (Fig. 2, A, C, and D; and Fig. S2 B). However, we could detect weak MT binding of the proline-rich part of the MAP7 linker as well as MAP7-Ct(mini) in COS7 cells, likely due to their flat morphology and low MT density (Figs. 2 D and S2 A). These data suggest that the C-terminal part of the MAP7 linker might have some weak MT affinity, which could contribute to but is unlikely to fully explain the ability of MAP7-Ct to rescue KIF5B-dependent mitochondria localization.

### K560 binding to MTs in cells depends on MAP7 proteins

**Figure 3.**
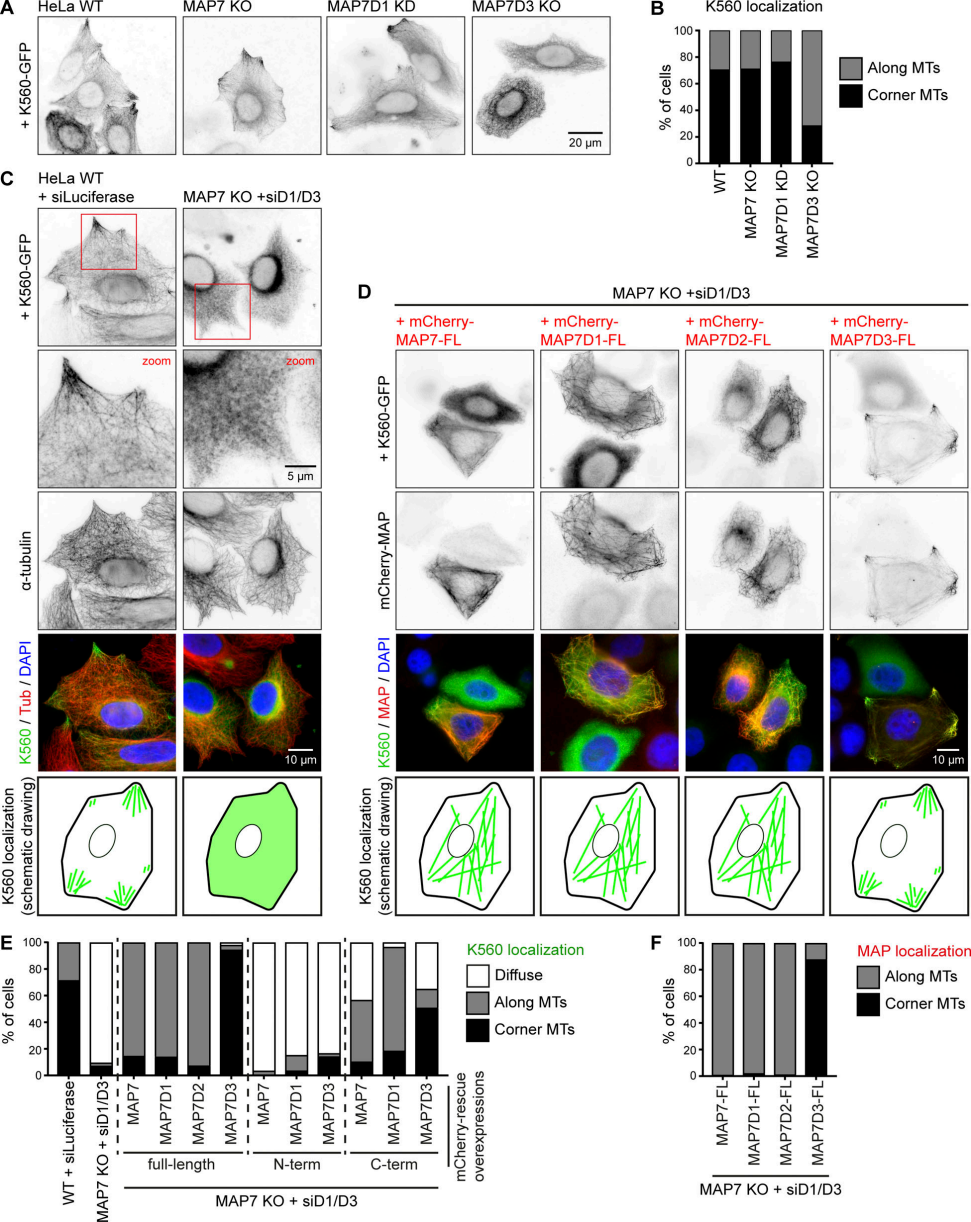
**Kinesin-1 recruitment to MTs depends on MAP7 family proteins. (A and B)** Widefield images of K560-GFP overexpressed in the indicated HeLa control, knockout (KO), or knockdown (KD) conditions (A) and quantification of K560-GFP localization (B). *n* = 234, 252, 174, and 254 cells from three independent experiments. **(C and D)** Widefield images of K560-GFP overexpressed either alone or together with mCherry-tagged MAP7 constructs in control or MAP7 KO + siMAP7D1/D3 HeLa cells, as indicated. In C, cells were costained for α-tubulin. A schematic drawing of K560-GFP localization is shown at the bottom. **(E and F)** Quantification of K560-GFP (E) and MAP7 construct (F) localization per condition, as indicated, categorized as diffuse, along MTs, or at corner MTs. *n* = 459 (WT + siLuciferase), *n* = 485 (MAP7 KO + siMAP7D1/D3), and for rescue conditions, *n* = 113, 237, 90, 167, 210, 186, 133, 176, 197, and 193 cells from two to four independent experiments.

To show that the loss of MAP7 proteins has a direct effect on kinesin-1 activity, we next examined the distribution of the dimeric K560 truncation mutant that can move along MTs but does not bind to cargo. In control HeLa cells, this construct was distributed along MTs, and in most cells, it showed enhanced accumulation on MTs in cell corners, where MT plus ends are concentrated (Fig. 3 A). Depletion of individual MAP7 family members did not alter this distribution except for the knockout of MAP7D3, in which less K560 accumulated on corner MTs (Fig. 3, A and B). In contrast, in cells lacking all three MAP7 proteins, K560 showed a diffuse localization (Fig. 3, C and E). Expression of MAP7, MAP7D1, or MAP7D2 in such cells rescued the recruitment of the kinesin to MTs, whereas expression of MAP7D3 led to strong coaccumulation of both constructs on MTs in the corners of almost all transfected cells (Fig. 3, D–F). Expression of the N-terminal, MT-binding fragments of MAP7 and its homologues could not restore the distribution of K560, whereas significant rescue of MT binding by the kinesin was observed with the C termini of all MAP7 proteins (Figs. 2 E and S2 B). We conclude that K560 displays low binding to cellular MTs in the absence of MAP7 and that this binding can be increased by kinesin-1–interacting MAP7-Ct fragments, which are diffusely localized in HeLa cells. The C terminus of MAP7D3, which was mostly nuclear on its own (Fig. S1 F), was retained in the cytoplasm when expressed together with K560, and shifted the localization of this kinesin fragment to MTs in cell corners, similar to the full-length MAP7D3 (Fig. 3, D and E; and Fig. S2 B).

### MAP7D3, but not MAP7, can be redistributed by kinesin-1

**Figure 4.**
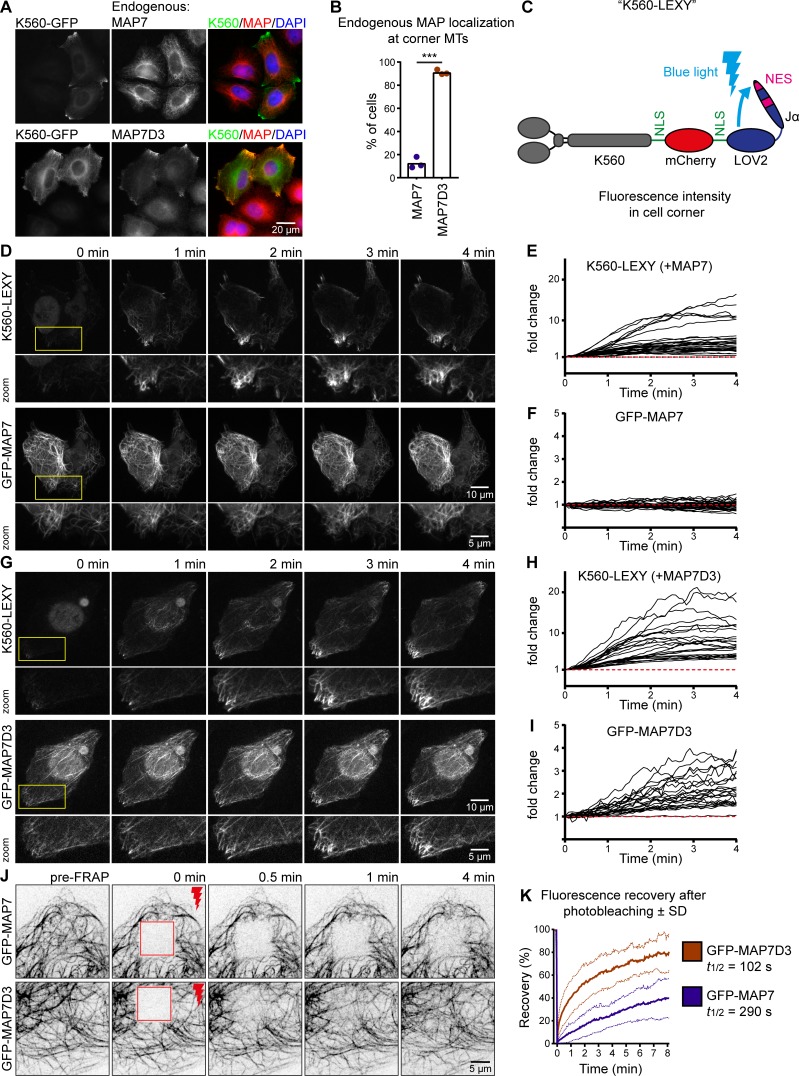
**Kinesin-1 can redistribute MAP7D3 in cells. (A and B)** Widefield images of K560-GFP overexpressed in HeLa cells stained for endogenous MAP7 or MAP7D3 (A) used to quantify endogenous MAP localization (B). *n* = 232 and 303 cells from three independent experiments, P < 0.001, *t* test. **(C)** Scheme of the K560-LEXY construct containing two NLS sequences and an mCherry tag. Blue light induces a conformation change, causing detachment of the Jα-peptide containing a nuclear export signal from the LOV2 domain. **(D and G)** Single frames of KIF5B KO cells cotransfected with K560-LEXY and GFP-MAP7 (D) or GFP-MAP7D3 (G) sequentially illuminated with green and blue light (in that specific order). Zooms are indicated in yellow. **(E, F, H, and I)** Measurements of fluorescence intensity changes over time in K560-LEXY–positive cell corners. Black lines represent single measurements of K560-LEXY (E and H), GFP-MAP7 (F), and GFP-MAP7D3 (I). *n* = 31 measurements from 17 cells (E and F) and *n* = 22 measurements from 14 cells (H and I) from two independent experiments. **(J)** Single frames of FRAP experiments on COS7 cells overexpressing GFP-MAP7 or -MAP7D3. Stills show a baseline (pre-FRAP), the first frame after photobleaching (0 min) and the indicated time points after FRAP of a 10 × 10-µm-square region (shown in red). **(K)** Quantification of fluorescence recovery of J. The graph shows mean curves (bold lines) ± SD (light dotted lines) over time. *n* = 18 cells from three independent experiments for each condition.

To understand why MAP7D3, but not the other MAP7 homologues, promotes MT plus-end–shifted distribution of K560, we next examined the distribution of endogenous MAP7 and MAP7D3 and found that only MAP7D3 could be efficiently relocalized by K560 to cell corners (Fig. 4, A and B).

To prove that kinesin-1 can indeed rapidly relocalize MAP7D3, we have set up an optogenetics-based assay in which K560 could be sequestered in the nucleus and then acutely released from it using a light-inducible nuclear export system (Niopek et al., 2016). A K560-mCherry, containing NLS sequences, was C-terminally tagged with an engineered domain of *Avena sativa* phototropin-1, AsLOV2, in which the Jα helix was modified to contain a nuclear export signal (Fig. 4 C). Within 1 to 2 min after activation with blue light, K560 was efficiently exported from the nucleus (Fig. 4, D, E, G, and H). MAP7D3, but not MAP7 coaccumulated on MTs in cell corners within a 4-min time frame (Fig. 4, D–I; and Videos 1 and 2). We conclude that K560 can indeed acutely relocalize its own positive regulator MAP7D3, but not MAP7, when kinesin expression is sufficiently high.

To explain why the distribution of MAP7D3, but not that of MAP7, was sensitive to the presence of kinesin-1, we hypothesized that MAP7D3 might be more mobile on MTs. To test this idea, we performed FRAP experiments with GFP-tagged MAP7 and MAP7D3 and found that the latter indeed exchanged much more rapidly on MTs (Fig. 4, J and K). The different turnover rates of the two MAP7 family proteins on MTs, possibly combined with the different affinities to kinesin-1, seem to contribute to their differential relocalization by overexpressed kinesin-1.

### MAP7 and MAP7D3 control kinesin-1 recruitment to MTs and motor processivity

To get further insight into the similarities and differences in the regulation of kinesin-1 by MAP7 proteins, we set up in vitro reconstitution assays. In contrast to previously published experiments, which employed Taxol-stabilized MTs in the absence of free tubulin, we used dynamic MTs that were grown from guanylyl-(α,β)-methylenediphosphonate (GMPCPP)–stabilized seeds (Bieling et al., 2007). Kinesins, MAPs, and MTs were observed by total internal reflection fluorescence microscopy, as described previously (van Riel et al., 2017). To study kinesin-1 motility, we purified full-length KIF5B-GFP and K560-GFP from HEK293T cells (Fig. S3 A). Analyses by mass spectrometry and Western blotting showed that although some copurification of MAP7, MAP7D1, and MAP7D3 with this kinesin was observed when the protein was washed with a low-ionic-strength buffer; this contamination was almost entirely removed when the ionic strength of the washing buffer was increased (Fig. S3, B–E). We used such “high-salt washed” kinesin preparations for all our experiments. In cells, kinesin-1 can exist in a heterotetrametric form with two light and two heavy chains. Although some light chains could be detected by Western blotting (Fig. S3 B), we assume that in our assays most full-length kinesins were dimers of heavy chains, as no light chains were visible on a Coomassie-stained gel (Fig. S3 A, arrowhead).

**Figure 5.**
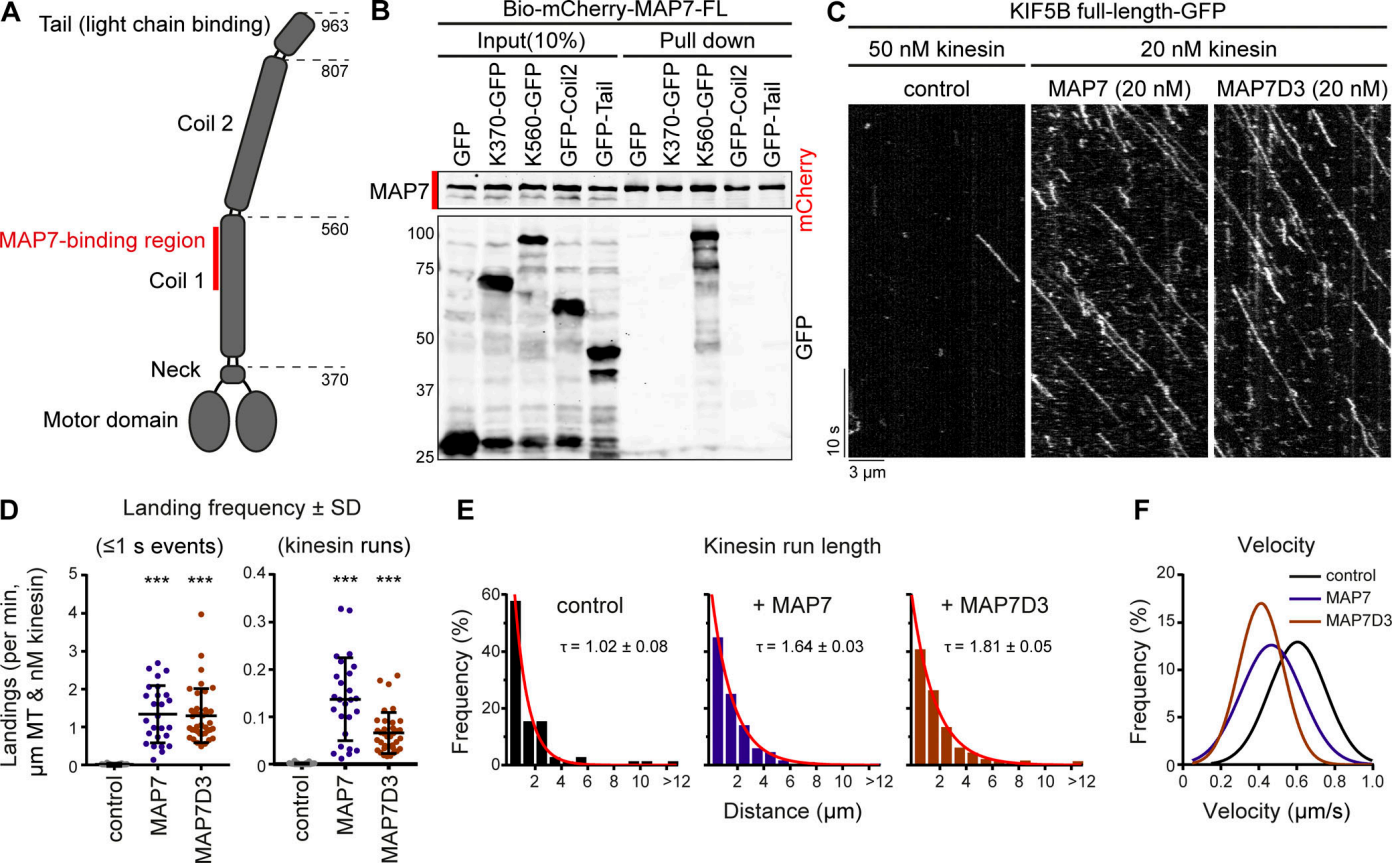
**Kinesin-1 is regulated by MAP7 proteins in vitro. (A)** Overview of full-length kinesin-1 (KIF5B) domains. **(B)** Streptavidin pull-down assay with extracts of HEK293T cells overexpressing BirA, the indicated KIF5B-GFP truncations (prey), and Bio-mCherry-MAP7 (bait) analyzed by Western blotting. **(C)** Kymographs of GFP-tagged full-length KIF5B (kinesin-1) on dynamic MTs in control conditions or in the presence of MAP7 or MAP7D3. **(D)** Quantification of kinesin-1 landing frequencies per MT and corrected for MT length, time of acquisition, and kinesin concentration. *n* = 99, 26, and 38 MTs from two or three independent experiments. **(E)** Histograms of run lengths fitted to a single exponential decay (red) with indicated rate constants (tau) as a measure of mean run length. *n* = 71, 542, and 568 kinesin runs from two or three independent experiments. **(F)** Gaussian fits of kinesin velocities. Histograms are shown in Fig. S3 G.

The MAP7-binding site on kinesin-1 is well defined (Monroy et al., 2018), and pull-down assays confirmed that there are no additional binding sites for MAP7 in the C-terminal coil or the tail of kinesin-1 (Fig. 5, A and B). Kymograph analysis of full-length kinesin-1 motors showed two populations of single-molecule behavior: short binding events that did not result in processive movement (classified as events that last ≤1 s) or events of landing followed by processive movement. Addition of purified Alexa Fluor 647–labeled SNAP-tag-MAP7 or -MAP7D3 (Fig. S3 A) in these assays led to a dramatic increase (at least 60-fold) of both types of events (Fig. 5, C and D). Furthermore, processive kinesin runs were on average ∼1.6-fold longer (Fig. 5, C and E), while the velocities were, especially upon addition of MAP7D3, reduced (Fig. 5, C and F; and Fig. S3 G).

**Figure 6.**
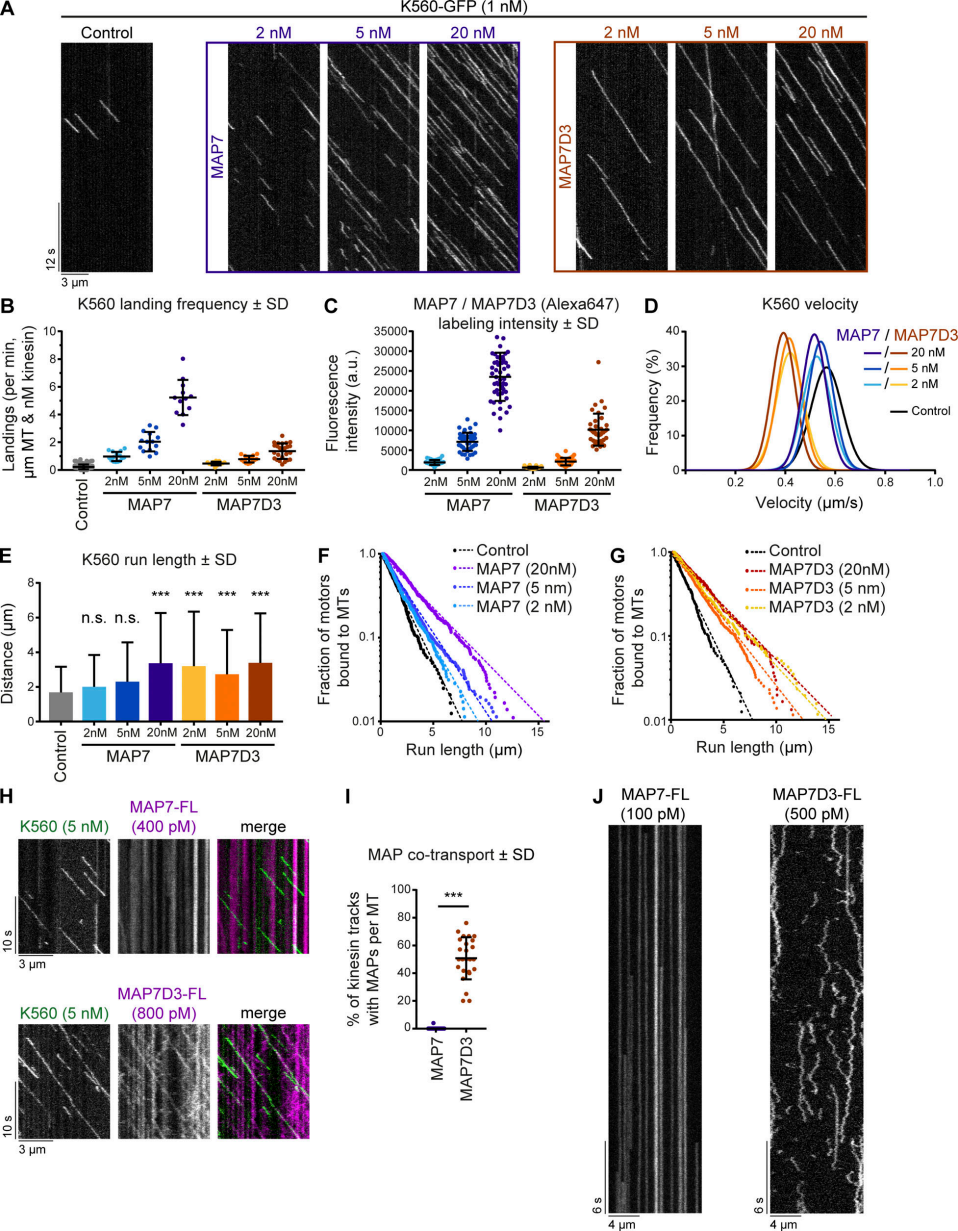
**Density and mobility of MAP7 determine kinesin-1 landing and processivity. (A)** Kymographs of K560-GFP on dynamic MTs in control conditions or in the presence of increasing concentrations of MAP7 or MAP7D3. **(B)** Quantification of kinesin landing frequencies per MT and corrected for MT length, time of acquisition, and kinesin concentration. *n* = 167, 15, 14, 12, 13, 15, and 25 MTs from two independent experiments. **(C)** Quantification of SNAP(Alexa Fluor 647)–tagged MAP7 and MAP7D3 intensities on dynamic MTs using images acquired under identical conditions on a TIRF microscope. *n* = 39 to 49 MTs from two independent experiments. Representative images are shown in Fig. S4 A. **(D)** Gaussian fits of kinesin velocities. Histograms are shown in Fig. S4 B. **(E)** Quantification of K560-GFP run length. *n* = 241, 351, 614, 361, 257, 436, and 303 kinesin runs from two independent experiments. ***, P < 0.001, Mann–Whitney *U* test. **(F and G)** Cumulative distributions of K560-GFP run lengths measured in the presence of increasing concentrations of MAP7 (F) or MAP7D3 (G). Straight dashed lines correspond to single exponential fits, n numbers correspond to E. **(H)** Kymographs of dual-color in vitro reconstitution experiments with K560-GFP and SNAP(Alexa Fluor 647)–tagged MAP7 or MAP7D3. **(I)** Quantification of kinesin tracks positive for MAP7 or MAP7D3 cotransport; *n* = 417 from 19 MTs (MAP7) and *n* = 344 from 25 MTs (MAP7D3) from two independent experiments. ***, P < 0.001, Mann–Whitney *U* test. **(J)** Kymograph of a single SNAP(Alexa Fluor 647)–tagged MAP7 or -MAP7D3 molecule on dynamic MTs in vitro. Videos were acquired at 25 frames/s on a TIRF microscope.

To further examine the effects of MAP7 and MAP7D3 on kinesin-1, we turned to the K560 fragment, which contains the MAP7-binding site but lacks the cargo-binding and autoinhibitory tail. When K560 was added to MAP7 or MAP7D3-decorated MTs, we observed a strong (up to 23.6-fold) increase in the motor landing frequency compared with K560 alone (Fig. 6, A and B), in agreement with published data on MAP7 (Monroy et al., 2018). The landing frequency of K560 increased with higher MAP concentrations and correlated with increasing MT labeling intensity by the particular MAP (Fig. 6, A–C; and Fig. S4 A). Furthermore, we found that MAP7D3, but not MAP7, caused a very significant decrease in kinesin velocity (Figs. 6 D and S4 B). Finally, we found that both MAP7 and MAP7D3 could induce a twofold increase in kinesin processivity (Fig. 6, E–G), with some kinesin runs exceeding 10 µm in length. We note that for this quantification, we only took into account the runs, in which we observed both kinesin association and dissociation from the MT. Inclusion of all detected runs suggested that in the presence of MAP7 or MAP7D3, even longer runs could occur (data not shown).

The increase of run lengths in the presence of MAP7 and MAP7D3 could be explained by kinesin multimerization or by a model where MAP7 acts as an additional MT attachment point. In these cases, the distribution of run lengths is expected to be described by the sum of two or three exponential decays (Klumpp and Lipowsky, 2005). However, the corresponding best fit of distributions in Fig. 6 (F and G) converged to a single exponential decay, suggesting that MAP7 directly affects kinesin’s binding–unbinding rate constants instead of introducing an additional intermediate binding state. Moreover, single-molecule analysis of K560 moving on MTs showed that kinesin-1 intensity profiles matched that of a single dimer in assays both with and without MAP7D3 (Fig. S4 C). In addition, we performed mixed kinesin assays where GFP- and SNAP(Alexa Fluor 647)-tagged kinesins were used in a 1:1 ratio. If kinesin-1 would multimerize in the presence of MAP7 proteins, then one would expect to see a significant fraction of two-colored kinesin tracks per kymograph; however, such events were not observed (Fig. S4 D), confirming our observation of K560 behaving as a single dimer on MAP7-decorated MTs. Taken together, these data suggest that the presence of MAP7 alters the state of single kinesin dimers.

Interestingly, MAP7 could promote kinesin processivity at high concentrations, when MTs were fully decorated, whereas MAP7D3 reduced kinesin detachment from MTs even at low concentrations (Fig. 6, C and E–G). These data correlated with the observation that MAP7D3, but not MAP7, could move together with K560 in vitro (Fig. 6, H and I). To find explanation for this difference in cotransport, we examined single-molecule dynamics of MAP7 and MAP7D3 on in vitro polymerized MTs and found that MAP7 showed very long static binding events, many of which exceeded our observation time (5 min; Fig. 6 J), in agreement with recently published data (Monroy et al., 2018). In contrast, MAP7D3 displayed a diffusive behavior, with many short binding events (Fig. 6 J). These data are in agreement with the FRAP data, showing that in cells, MAP7D3 is more mobile than MAP7 (Fig. 4, J and K). The density of MT labeling was higher with MAP7 than with MAP7D3 at the same protein concentration, indicating that the latter has a lower affinity for MTs (Fig. 6, C and J; and Fig. S4 A). We conclude that MAP7 proteins can affect not only kinesin landing on MTs, as suggested previously (Sung et al., 2008; Monroy et al., 2018), but also its processivity. Cotransport of the MAP with the kinesin could facilitate processive motion but was not essential, as a statically bound MAP7 could also exert this effect if its density on MTs was high enough.

### MAP7D3-Ct promotes MT recruitment and processivity of kinesin-1 in spite of having a low MT affinity

**Figure 7.**
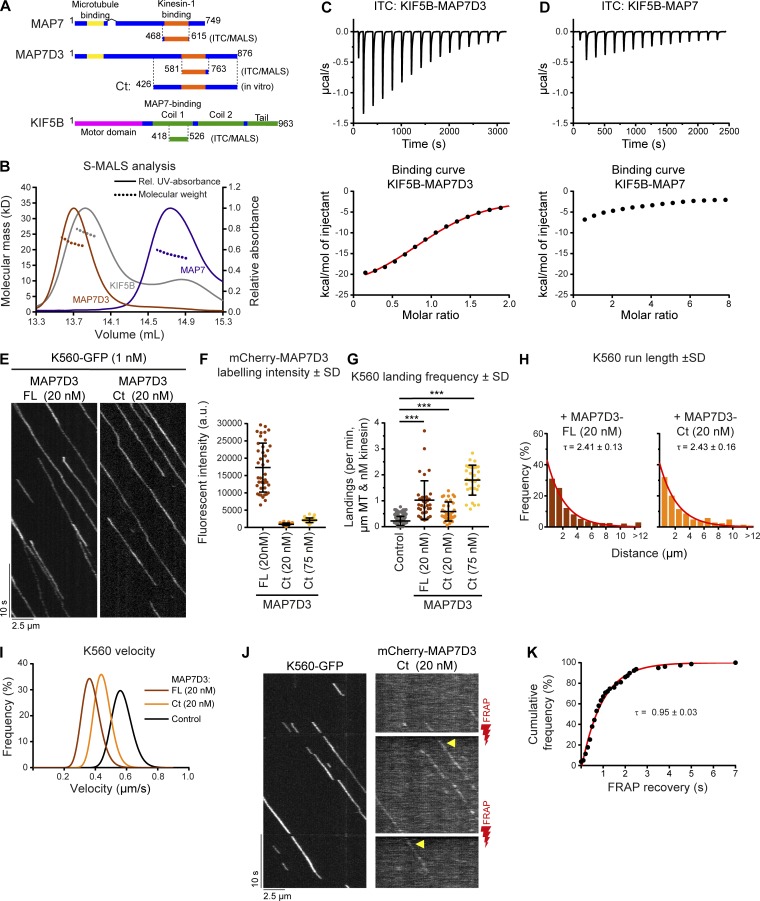
**MAP7D3 binds to KIF5B more tightly than MAP7 and promotes its activity in vitro. (A)** Schemes of MAP7 family proteins and KIF5B constructs used in this figure. **(B)** SEC-MALS was used to determine the oligomerization state of indicated proteins in solution. Lines depict the relative UV-absorbance measured at 280 nm plotted against the elution volume. The molecular mass determinations by multi-angle light scattering are depicted by dashed lines. **(C and D)** ITC of MAP7D3 (C) or MAP7 (D) against KIF5B. Top: Enthalpograms of the respective titrations. Bottom: integrated heat change (black dots) and the associated fitted curve (red line in C). Controls are shown in Fig. S4 (E–G). **(E)** Kymographs of K560-GFP on dynamic MTs in the presence of full-length (FL) MAP7D3 or MAP7D3-Ct (both purified from *E. coli*). **(F)** Quantifications of MAP7D3-FL or -Ct intensities on dynamic MTs using images acquired under identical conditions on a TIRF microscope. *n* = 40, *n* = 38, and *n* = 42 MTs from two independent experiments. Representative images are shown in Fig. S5 A. **(G)** Quantification of landing frequency per MT and corrected for MT length, time of acquisition, and kinesin concentration. *n* = 167, 36, 36, and 31 MTs from two independent experiments. **(H)** Histograms showing kinesin run lengths fitted to a single exponential decay (red) with the indicated rate constants (tau) as a measure of mean run length; *n* = 241, 271, and 209 kinesin runs from two independent experiments. The associated bar plots are shown in Fig. S5 C. **(I)** Gaussian fits of kinesin velocities. Histograms are shown in Fig. S5 B. **(J)** Kymographs of single-molecule FRAP experiments on K560-GFP motors and MAP7D3-Ct. Photobleaching with a 561-nm red laser was performed at time points indicated by red lightning bolts. Fluorescent recovery is indicated by arrowheads. **(K)** Cumulative frequency distribution plot of mCherry-MAP7D3-Ct recovery after photobleaching (black dots) fitted to an exponential decay (red line) with the indicated decay constant (tau); *n* = 79 from three independent experiments.

The ability of K560 to transport MAP7D3, but not MAP7, might be caused not only by the different MT-binding behavior of the two MAPs but also by their different affinities for the kinesin. To test this possibility, we purified minimal binding constructs for MAP7, MAP7D3, and KIF5B from *Escherichia coli* (Figs. 7 A and S3 F) and probed the oligomerization state of the individual proteins in solution by size exclusion chromatography followed by multiangle light scattering (SEC-MALS). As expected, both the MAP7 and MAP7D3 fragments were monomers with the measured molecular mass values of 18.4 kD (calculated molecular mass of 18.8 kD) and 22.2 kD (calculated molecular mass of 21.5 kD), respectively, whereas the KIF5B fragment was a dimer (measured and calculated molecular mass of 25.9 kD; Fig. 7 B). Using isothermal titration calorimetry (ITC) experiments, we found an equilibrium dissociation constant, *K*_d_, of 3.8 ± 0.2 µM and a stoichiometry number, N, of 0.96 (monomer equivalents) for the interaction between the KIF5B and MAP7D3 fragments (Fig. 7 C and Fig S4, E and F). In contrast, the MAP7 fragment had an affinity for the KIF5B fragment that was too weak to be properly determined; however, the obtained isotherm suggested a *K*_d_ in the higher micromolar range (Fig. 7 D and Fig. S4, E and G).

Since MAP7D3 seems to be the most potent kinesin-1 interactor, we set out to compare the effect of full-length MAP7D3 and its C terminus (also used for cellular experiments), both purified from *E. coli* (Figs. 7 A and S3 F), on K560 motility in vitro (Fig. 7 E). In agreement with a previous publication (Yadav et al., 2014), MAP7D3-Ct displayed weak MT binding: MT labeling intensity with 20 nM MAP7D3-Ct was 19.2-fold lower than with 20 nM full-length MAP7D3 (Figs. 7 F and S5A). In spite of this lower MT affinity, MAP7D3-Ct could efficiently increase K560 landing rate, promote its processivity, and decrease motor velocity compared with control (Fig. 7, E–I; and Fig. S5, A–C). The effect of MAP7D3-Ct on the landing rate was particularly obvious at 75 nM concentration, as MT labeling at this concentration was still 8.3-fold lower than with 20 nM full-length MAP7D3, whereas the K560 landing frequency was 8.1-fold higher compared with control (kinesin only; Fig. 7, F and G). These data argue against the simple model that MAP7D3 acts as MT-recruiting factor for kinesin-1 but cannot exclude that the weak binding of MAP7D3-Ct to MTs augments K560-MT interaction. These data also show that the addition of a weak MAP module to the kinesin coil is sufficient to make kinesin-1 processive.

Simultaneous imaging of K560-GFP and mCherry-MAP7D3-Ct showed that this truncated MAP colocalized with moving kinesins (Fig. 7 J). Importantly, FRAP analysis in the mCherry channel, leaving the GFP fluorescence unaffected, showed that MAP7D3-Ct exchanged rapidly on moving K560 motors (Fig. 7, J and K). Together with the micromolar-range *K*_d_ of the kinesin–MAP binding (Fig. 7, C and D), these data support the idea that fast binding–unbinding kinetics enables static MAP7 proteins to promote processive movement of kinesin-1.

### MAP7-Ct promotes kinesin-1 landing on MTs independently of MT tethering

**Figure 8.**
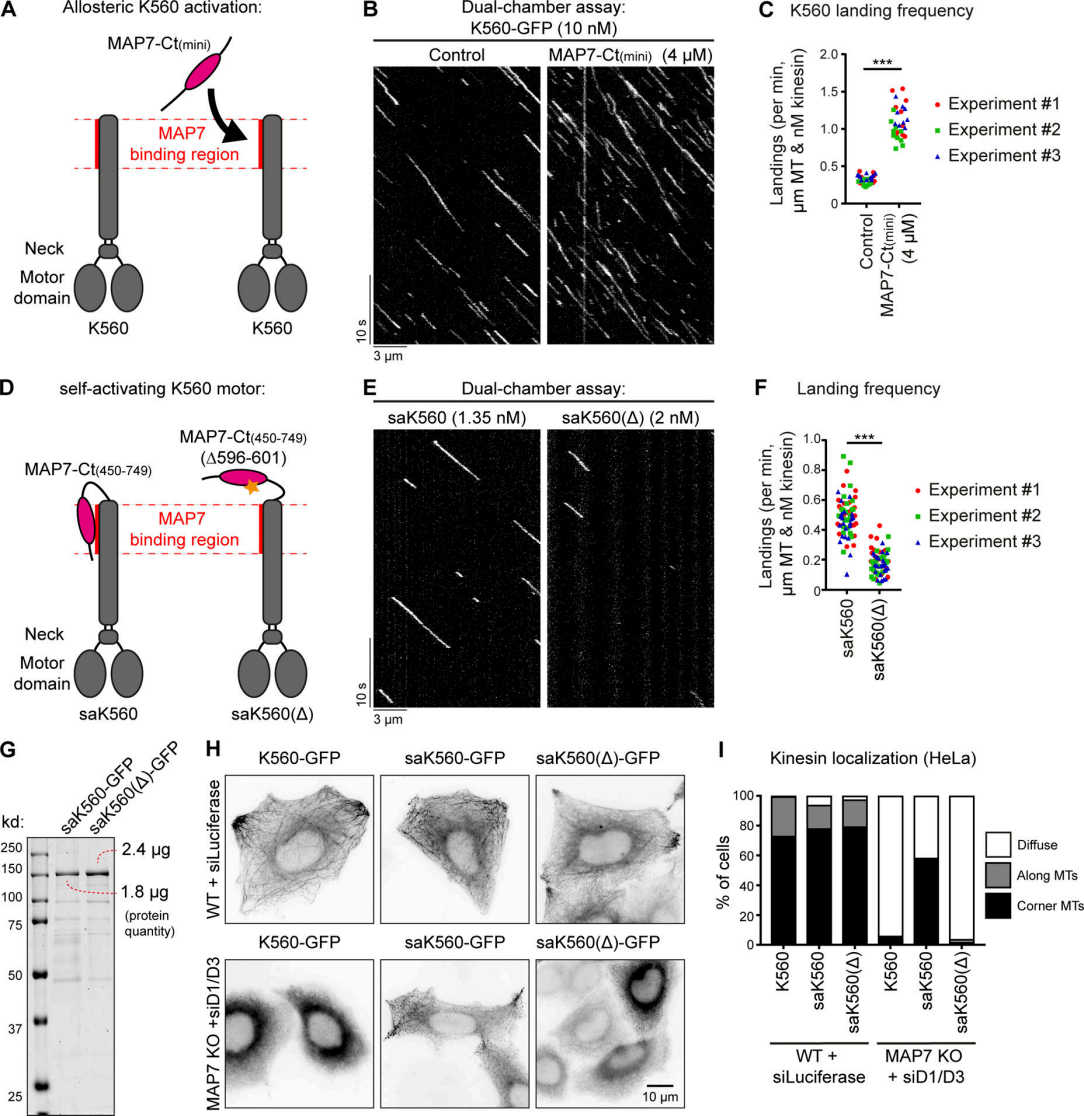
**MAP7-Ct can activate kinesin-1. (A and D)** Overview of kinesin-1 and MAP7 constructs used for experiments. **(B and E)** Kymographs of a dual-chamber in vitro experiment, where equal concentrations of K560-GFP motors were added to chambers with or without MAP7-Ct(mini) (B), or with the indicated concentrations of saK560/saK560(Δ) (E) on dynamic MTs. **(C and F)** Landing frequencies quantified per MT and corrected for MT length, time of acquisition, and kinesin concentration. Each independent dual-chamber experiment is color-coded; *n* = 32 and 29 MTs (C) and *n* = 73 and 69 MTs (F), all from three independent experiments. ***, P < 0.001, Mann–Whitney *U* test. **(G)** Analysis of purified saK560 proteins by SDS-PAGE. Protein concentrations were determined from a single gel using BSA standard. **(H)** Widefield images of overexpressed GFP-tagged kinesin constructs in control or MAP7 KO + siMAP7D1/D3 HeLa cells. **(I)** Quantification of kinesin localization categorized as diffuse, along MTs, or at corner MTs. *n* = 203, 172, 198, 241, 186, and 234 cells from three independent experiments.

To investigate whether MAP7 family proteins can exert an effect on kinesin-1 that is independent of MT tethering, we used MAP7-Ct(mini) (Fig. 8 A), which was completely diffuse in HeLa cells, showed only very weak MT binding in COS7 cells (Fig. 2 A, C, and D; and Fig. S2 A), and could be prepared from *E. coli* at high concentration in an untagged form (Fig. S3 F). When added at micromolar concentrations to the assay with K560, this protein fragment caused a significant (3.4-fold) increase in the motor landing frequency (Fig. 8, B and C), whereas the velocity of the kinesin was only mildly affected (Fig. S5 E). Strikingly, the increase in motor processivity observed with the MAP7D3-Ct was not detected with MAP7-Ct(mini) (Fig. S5, F and G).

To further substantiate the observation that the MAP7-Ct can activate kinesin-1 independently of any residual MT affinity, we developed a self-activating K560 motor, saK560, where the kinesin-binding domain of MAP7 lacking both the P-region and the proline-rich region was fused to the C terminus of K560. As a negative control, we generated a saK560(Δ) mutant lacking six amino acids of MAP7 essential for the kinesin binding (Fig. 8 D; Monroy et al., 2018). Comparison of these two kinesins showed that the saK560 had a higher landing frequency (Fig. 8, E–G), while other parameters such as velocity and run length were not much affected (Fig. S5, H–J). In further support of this observation, we examined the behavior of K560 together with the self-activating fusion proteins in HeLa cells and saw that they all behaved very similarly in wild-type cells (Fig. 8 H, top). However, upon depletion of all MAP7 family proteins, saK560(Δ) motor was diffuse, just like K560; however, the saK560 kinesin showed enhanced localization on MTs in cell corners (Fig. 8, H and I), demonstrating that this motor still displays activity despite of the absence of MAP7 family protein. Altogether, MT landing of kinesin-1 can be increased by MAP7 family proteins independently of their MT interaction, whereas the regulation of kinesin processivity by these MAPs depends on their association with MTs.

### The stalk of K560 inhibits MT interaction

**Figure 9.**
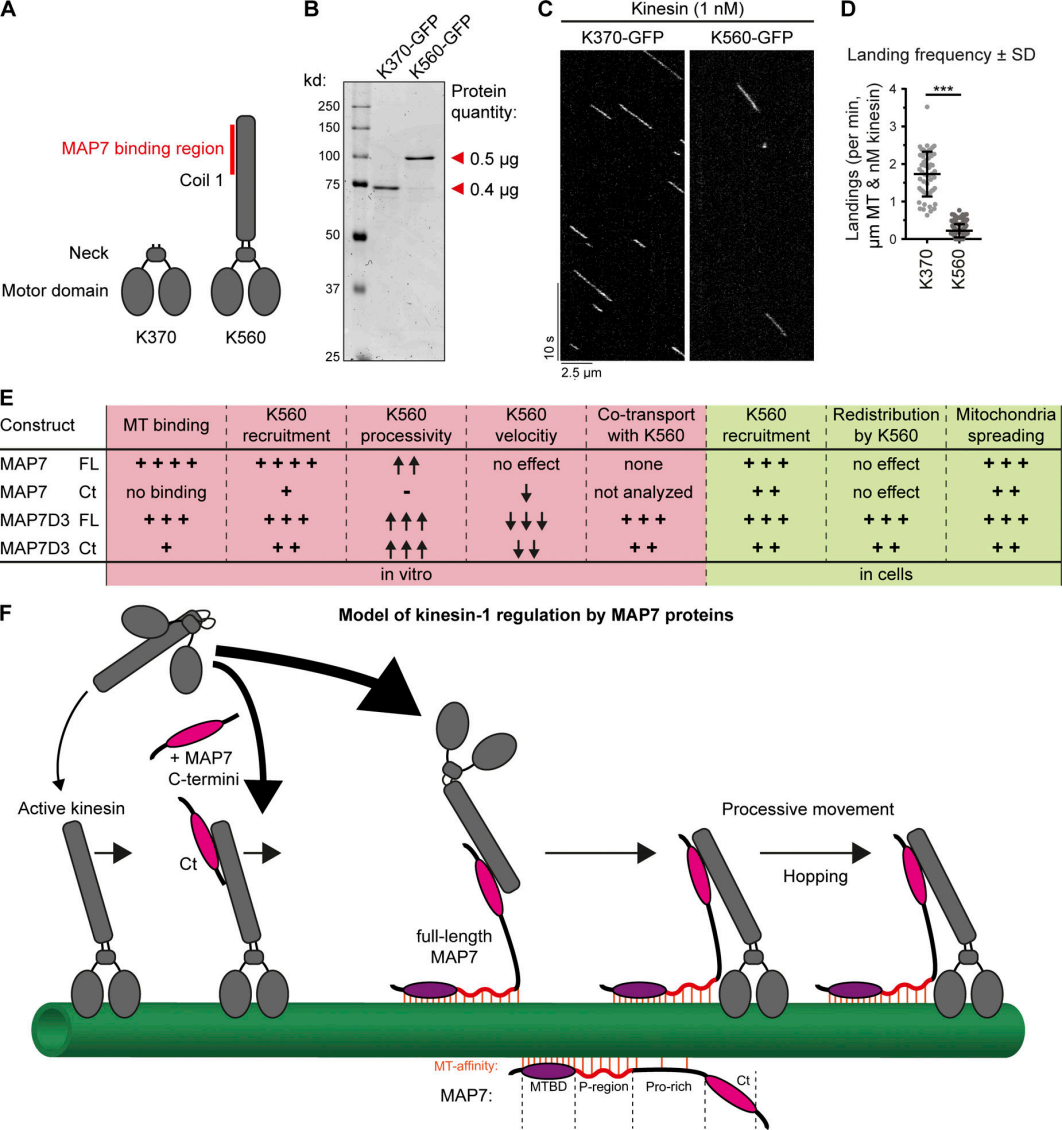
**Removal of the kinesin-1 stalk domain enhances motor landing. (A)** Overview of kinesin-1 constructs used. **(B)** Purified K370-GFP and K560-GFP analyzed by SDS-PAGE. Protein concentrations were determined using BSA standard. **(C)** Kymographs of K370-GFP and K560-GFP motors moving on dynamic MTs. **(D)** Quantification of kinesin landing frequencies; *n* = 57 (K370-GFP) from three independent experiments and *n* = 167 (K560-GFP) from two independent experiments. ***, P < 0.001, Mann–Whitney *U* test. **(E)** Summarizing table of the characteristics and effects of MAP7 proteins on different parameters of kinesin activity observed in vitro and in cells. **(F)** Working model of kinesin-1 activation by MAP7 family proteins. Red lines indicate MAP–MT interactions.

Our finding that MAP7-Ct improves the MT landing frequency of K560 could potentially be explained if the MAP7-interacting stalk of kinesin-1 partly interferes with MT binding. If this were true, then a kinesin-1 truncation lacking this stalk should bind to MTs more efficiently. To test this idea, we generated a shorter KIF5B truncation mutant, KIF5B 1–370 (K370), which lacks the MAP7-binding coil region but still dimerizes via its neck linker (Kozielski et al., 1997; Fig. 9, A and B; and Fig. S5 D). In in vitro assays, K370 appeared to be a faster kinesin with slightly shorter runs compared with K560 (Fig. 9 C and Fig. S5, K–M). Importantly, we observed a 7.8-fold difference in motor landing frequency (Fig. 9 D), indicating that the presence of the stalk region in K560 has a negative effect on its interaction with MTs.

## Discussion

In this study, we have systematically analyzed the impact of mammalian MAP7 family proteins on kinesin-1 transport (Fig. 9 E). We found that at least one MAP7 homologue was necessary and sufficient to enable kinesin-1–driven mitochondria distribution. These results are in agreement with the data showing that MAP7/ensconsin is an essential kinesin-1 cofactor in flies and in mammalian muscle cells (Sung et al., 2008; Metzger et al., 2012; Barlan et al., 2013; Metivier et al., 2018; Monroy et al., 2018). Dependence on MAP7 family members likely applies to many other kinesin-1–driven processes in mammals, because the core part of kinesin-1, the K560 fragment, was unable to bind to MTs efficiently in cells when all MAP7 homologues were absent.

Kinesin-1 function could be rescued to a significant extent by a MAP7 fragment that binds to kinesin but has only low MT affinity, again in agreement with the data obtained in *Drosophila melanogaster* (Barlan et al., 2013; Metivier et al., 2018). K560 recruitment to MTs was also stimulated by this MAP7 fragment both in cells and in vitro. This effect can likely be explained by a combination of two factors: the weak residual MT affinity present in the unstructured part of MAP7-Ct (proline-rich region) as well as an allosteric regulation of the K560 motor by the kinesin-binding domain of MAP7. The ability of MAP7 to affect K560 allosterically is supported by the increased landing rate of the “self-activating” KIF5B–MAP7 fusion, which lacks all MAP7 linker sequences with potential MT affinity. This notion is also supported by the observation that a short kinesin-1 version (K370) lacking the MAP7-binding stalk region interacts with MTs more efficiently than K560. We propose that the stalk-containing kinesin can adopt conformations that are unfavorable for MT binding, whereas the interaction with MAP7 allosterically stabilizes a conformation that promotes landing on MTs (Fig. 9 F). Diverse regulatory steps have been described for kinesin-1, mostly involving the autoinhibitory C-terminal tail region (Verhey and Hammond, 2009) and also the motor domain (Xu et al., 2012). Our data add to this complexity by showing that the state of the stalk and its binding partners might directly affect kinesin interaction with MTs.

Although a MAP7 fragment lacking MT affinity can promote the engagement of kinesin-1 with MTs, the presence of MT-binding regions makes this regulation much more efficient and robust. Without it, a very high concentration of the kinesin-binding domain or its direct fusion to kinesin was required to activate the motor. The presence of MT-binding sites concentrates the kinesin-binding domain of the MAP on MTs and can thus facilitate its interaction with the motor (Fig. 9 F). Importantly, MT-bound MAP7 proteins have a significant effect not only on the kinesin landing rate but also on its processivity, and we excluded the possibility that this was due to kinesin multimerization. Interestingly, both a very immobile MAP (MAP7) and a more dynamic and diffusively behaving MAP (MAP7D3) could enhance kinesin-1 processivity, but the latter was able to exert this effect when present on a MT at a lower density. An obvious hypothesis is that kinesin processivity is governed by the presence of additional links to MTs. However, the distribution of run lengths of K560 was monoexponential even in the presence of MAP7 proteins, whereas a significant contribution of an additional MAP-dependent MT bound state would be expected to lead to a distribution corresponding to the sum of two or three exponential decays (Klumpp and Lipowsky, 2005). These data suggest that the interaction with MT-bound MAP7s does not create an additional connection to a MT but somehow alters the conformational state of the motor to prevent its dissociation from MTs. Additional work would be needed to understand why a MT-unbound MAP7 fragment is sufficient to promote kinesin-1 landing but does not increase its processivity even when directly fused to the motor, whereas a MT-bound MAP7 version can stimulate not only the initial kinesin engagement with a MT but also its processive motility.

The relatively low affinity of the MAP–kinesin interaction and the rapid binding–unbinding kinetics suggest that the kinesin could be “hopping” from one MAP molecule to another, and this can allow even a highly immobile MAP7 to counteract kinesin dissociation without strongly affecting motor velocity. MAP7D3 is different from MAP7 because it binds less tightly to MTs and more tightly to the kinesin and can be “dragged” with the motor to some extent. This possibly explains why it slows down kinesin movement and why its low concentration is sufficient to increase motor processivity. However, because of the fast turnover within the MAP–motor complex, MAP7D3 is unlikely to be undergoing large-distance transport by the kinesin in cells. MAP7D3 can be rapidly relocalized to MT plus ends by overexpressed K560, but at the endogenous kinesin-1 expression levels, MAP7D3 is not enriched at MT plus ends, suggesting that the levels of endogenous motor are insufficient to drive MAP7D3 to the cell periphery. Still, it is possible that also the endogenous kinesin-1 could cause some redistribution of its own positive regulators, which would be in line with some previous observations (Kikuchi et al., 2018).

Recent work showed that MAP7 is a powerful MT recruiter of kinesin-1 (Monroy et al., 2018; Tymanskyj et al., 2018). Here, we confirmed these observations and provided insight into the underlying molecular mechanism. In addition, we showed that other MAP7 family members can act redundantly but also possess unique properties that could fine-tune kinesin-1 regulation. Support for the latter idea can be found from the work in neurons, where it was shown that several individual MAP7 proteins, such as MAP7 and MAP7D1, are involved in cargo transport and neurite development (Sung et al., 2008; Barlan et al., 2013; Koizumi et al., 2017; Tymanskyj et al., 2017, 2018; Monroy et al., 2018). It is therefore an intriguing question whether specific combinations of MAP7 proteins could provide spatiotemporal control of kinesin transport in complex cell types and whether, as has been shown for MAP7 (Monroy et al., 2018), this involves competition with other MAPs or modulation of other transporting motors. Another unanswered question is whether the localization of MAP7 proteins contributes to the well-documented selectivity of kinesin-1 for specific MT tracks, which appear to correspond to the stable, long-lived MT populations (Nakata and Hirokawa, 2003; Jacobson et al., 2006; Hammond et al., 2008; Cai et al., 2009; Farías et al., 2015; Guardia et al., 2016; Tas et al., 2017). MAP7, which is stably associated with MTs, could potentially predispose kinesin-1 for interacting with more long-lived MTs, on which this MAP would gradually accumulate. Taken together, our data illustrate the complexity of the interplay between the motors and the tracks they use during intracellular transport processes.

## Materials and methods

### Cell culture, knockdowns, and CRISPR-Cas9 knockouts

HeLa (Kyoto), COS7, and human embryonic kidney 239T (HEK293T) cell lines were cultured in medium that consisted of 45% DMEM, 45% Ham’s F10, and 10% fetal calf serum supplemented with penicillin and streptomycin. The cell lines were routinely checked for mycoplasma contamination using LT07-518 Mycoalert assay (Lonza). HeLa and COS7 cells were transfected with plasmids using FuGENE 6 (Promega) for generating knockout lines, live-cell imaging, and immunofluorescence experiments. For streptavidin pull-down assays and protein purification from HEK293T cells, plasmids were transfected with polyethylenimine (Polysciences). For generating knockdowns, HeLa cells were transfected with 100 nM siRNA for each target using HiPerfect (Qiagen). The following siRNAs were used in this study: MAP7D1 (target sequence 5′-TCA​TGA​AGA​GGA​CTC​GGA​A-3′), MAP7D3 (target sequence 5′-AAC​CTA​CAT​TCG​TCT​ACT​GAT-3′), and luciferase (target sequence 5′-TCG​AAG​TAT​TCC​GCG​TAC​G-3′). For rescue experiments, HeLa cells were transfected 2 d after siRNA transfection.

HeLa CRISPR-Cas9 knockout lines were generated using the pSpCas9-2A-Puro (PX459) vector, purchased from Addgene (Ran et al., 2013). Guide RNAs for human KIF5B, MAP7, and MAP7D3 were designed using the CRISPR design webpage tool (http://crispr.mit.edu). The targeting sequences for gRNAs were as follows (coding strand sequence indicated): KIF5B, 5′-CCG​ATC​AAA​TGC​ATA​AGG​CT-3′; MAP7, 5′-CGC​CCT​GCC​TCT​GCA​ATT​TC-3′; and MAP7D3, 5′-CCG​TGC​CCG​CAG​CTC​TCT​CA-3′. The CRISPR-Cas9–mediated knockout of KIF5B, MAP7, and MAP7D3-encoding genes was performed according to the protocol described in Ran et al. (2013). In brief, HeLa cells were transfected using Fugene6 (Promega) with the vectors bearing the appropriate targeting sequences. Cells were subjected to selection with 2 µg/ml puromycin 24–48 h after transfection for 48–72 h. After selection, cells were allowed to recover in complete medium for ∼7 d; meanwhile, cells were diluted in 96-well plates for growing single-cell colonies. Knockout efficiency was controlled using mixed cell populations by immunofluorescence staining, and depending on the efficiency, 5–30 individual clones were isolated and characterized by Western blotting and immunostaining.

### DNA constructs

All MAP7 family protein constructs except MAP7D1-FL were cloned by a PCR-based strategy into a pBio-mCherry-C1 vector, a modified pEGFP-C1 vector, in which the open reading frame encoding EGFP was substituted for mCherry, and a linker encoding the sequence MASGLNDIFEAQKIEWHEGGG, which is the substrate of biotin ligase BirA, was inserted into the NheI and AgeI sites in front of the mCherry-encoding sequence. MAP7D1-FL has been cloned into a Bio-mCherry-C3 vector in a similar fashion. MAP7 constructs were generated using cDNA (Faire et al., 1999), was a kind gift of C. Bulinski (Columbia University Medical Center, New York, NY) or cDNA of RNA isolated from HeLa cells. MAP7D1 was derived from the IMAGE clone 6514558 (Source Bioscience), MAP7D2 was derived from the IMAGE clone 3063898 (Source Bioscience), and MAP7D3 was from the IMAGE clone 5284128 (Source Bioscience). All MAP7 linker constructs were cloned in a pEGFP-C1 vector by a PCR-based strategy. Rescue constructs for MAP7D1 and MAP7D3 were obtained by PCR-based mutagenesis of the sequence 5′-TCA​TGA​AGA​GGA​CTC​GGA​A-3′ to 5′-TCA​TGA​AGA​GAA​CAC​GCA​A-3′ (MAP7D1) and 5′-AAC​CTA​CAT​TCG​TCT​ACT​GAT-3′ to 5′-AAT​CTA​CAC​TCG​TCT​ACA​GAT-3′ (MAP7D3). For protein purification from HEK293T cells, MAP7-Ct was cloned into a pmCherry-C1 vector with an N-terminal Strep-tag, and full-length MAP7 and MAP7D3 were cloned into a pTT5 vector with an N-terminal SNAP- and Strep-tag. For bacterial purifications, MAP7D3 constructs were cloned into a pET28a vector containing an N-terminal Strep-tag. MAP7-Ct(mini) construct was cloned by a PCR-based strategy into a pET24a vector containing an N-terminal SUMO-tag. All KIF5B constructs were cloned by a PCR-based strategy into pEGFP-N1 vectors. K560-GFP and full-length KIF5B-GFP used for protein purification were cloned into a pTT5 vector with a C-terminal Strep-tag. K370-GFP, saK560-GFP, and saK560-GFP(Δ) used for protein purification and cellular experiments were cloned into a pEGFP-N1 vector with a C-terminal Strep-tag. GFP-KIF5B-Coil2 and GFP-KIF5B-Tail were cloned in a pEGFP-C1 vector. All kinesin constructs were based on full-length human KIF5B as template IMAGE clone 8991997 (van Spronsen et al., 2013). KIF5B-mCherry-Lexy was cloned by PCR-based strategy. A c-myc NLS sequence was introduced between K560 and mCherry (amino acid sequence: PAAKRVKLD) and a second SV40 Large T-antigen NLS was introduced between mCherry and the LEXY domain (amino acid sequence: PKKKRKV). The engineered LOV2-domain (LEXY) for blue light–inducible nuclear export was obtained from Addgene (catalog number 72655; Niopek et al., 2016). For affinity measurements, minimal protein fragments for MAP7 and MAP7D3 were designed based on structure prediction and intron–exon analysis. For KIF5B, the designed fragment was the shortest construct still containing the cysteine at position 421 and the MAP7-binding domain (amino acids 508–523; Monroy et al., 2018). All protein constructs were fused to an N-terminal thioredoxin-6xHis cleavable tag by restriction free positive selection into a pET-based bacterial expression vector by a PCR-based strategy (Olieric et al., 2010). Human EB3-GFP is described previously (Stepanova et al., 2003). Biotin ligase BirA expression construct (Driegen et al., 2005) was a kind gift from D. Meijer (University of Edinburgh, Edinburgh, UK).

### Antibodies, Western blotting, and immunofluorescence cell staining

For immunofluorescence cell staining and Western blotting, we used rabbit polyclonal antibodies against MAP7D1 (HPA028075; Sigma-Aldrich/Atlas), MAP7D2 (HPA051508; Sigma-Aldrich/Atlas), MAP7D3 (HPA035598; Sigma-Aldrich/Atlas), kinesin heavy chain (UKHC H-50, SC28538; Santa Cruz), kinesin light chain-1 (H-75, SC25735; Santa Cruz) and GFP (ab290; Abcam). We used a mouse polyclonal antibody against MAP7 (H00009053-B01P; Abnova) and mouse monoclonal antibodies against Ku80 (611360; BD Bioscience), mCherry (632543; Clontech), cytochrome *c* (556432; BD Bioscience), and α-tubulin (T6199; Sigma-Aldrich), and a rat monoclonal antibody against α-tubulin (γL1/2, ab6160; Abcam). The following secondary antibodies were used: IRDye 800CW/680LT goat anti–rabbit, and anti–mouse for Western blotting and Alexa Fluor 488–, 594–, and 647–conjugated goat antibodies against rabbit, rat, and mouse IgG (Molecular Probes) for immunofluorescence. Mitotracker Red CMXRos (Molecular Probes) was alternatively used for mitochondria staining.

Total HeLa cell extracts were prepared in RIPA buffer containing 50 mM Tris-HCl, pH 7.5, 150 mM NaCl, 1% Triton X-100, 0.5% SDS, and cOmplete protease inhibitor cocktail (Roche).

For immunofluorescence cell staining, HeLa cells were fixed in −20°C methanol for 10 min and stained for MAP7, MAP7D1, MAP7D2, MAP7D3, and α-tubulin. In the case of cytochrome *c*, cells were fixed with 4% PFA in PBS for 10 min. Cells were then permeabilized with 0.15% Triton X-100 in PBS for 2 min; subsequent wash steps were performed in PBS supplemented with 0.05% Tween-20. Epitope blocking and antibody labeling steps were performed in PBS supplemented with 0.05% Tween-20 and 1% BSA. Before mounting in Vectashield mounting medium (Vector Laboratories), slides were washed with 70% and 100% ethanol and air-dried.

### Pull-down assays

Streptavidin pull-down assays were performed from HEK293T cell lysates by coexpressing biotin ligase BirA with mCherry-tagged constructs containing a biotinylation site (pBio-mCherry; bait) and GFP-tagged KIF5B constructs (prey). Constructs were transfected together into HEK293 cells using polyethylenimine with a 24-h incubation time for protein expression. M-280 Streptavidin Dynabeads (Invitrogen) were blocked in a buffer containing 20 mM Tris, pH 7.5, 20% glycerol, 150 mM NaCl, and 10 µg chicken egg albumin followed by three washes with wash buffer containing 20 mM Tris, pH 7.5, 150 mM NaCl, and 0.1% Triton X-100. HEK293T cells were scraped and collected in ice-cold PBS followed by lysis on ice in a buffer containing 20 mM Tris, pH 7.5, 150 mM NaCl, 1 mM MgCl_2_, 1% Triton X-100, and cOmplete protease inhibitor cocktail (Roche). To separate cell debris, the lysates were cleared by centrifugation at 4°C for 15 min at 16,000 *g*, and 10% of each lysate was saved as input control. Cell lysates were incubated with preblocked streptavidin beads for 60 min at 4°C followed by five washes with the buffer containing 20 mM Tris, pH 7.5, 150 mM NaCl, and 0.1% Triton X-100. Streptavidin beads were pelleted and boiled in 2× Laemmli sample buffer. Protein lysates and pull down of both bait and prey proteins were analyzed by Western blotting.

### Protein purification

All KIF5B, SNAP(Alexa Fluor 647)–labeled proteins and mCherry-MAP7-Ct used for in vitro reconstitution assays were purified from HEK293T cells using Strep(II)-streptactin affinity purification. Cells were harvested 24–40 h after transfection. Cells from a 15-cm cell culture dish were lysed in 800 µl lysis buffer containing 50 mM Hepes, pH 7.4, 300 mM NaCl, 1 mM MgCl_2_, 1 mM DTT, and 0.5% Triton X-100 supplemented with cOmplete protease inhibitor cocktail (Roche) on ice for 15 min. The supernatants obtained from cell lysates after centrifugation at 16,000 *g* for 20 min were incubated with 50 µl StrepTactin Sepharose beads (GE Healthcare) for 1 h The beads were washed five times in a high-salt wash buffer containing 50 mM Hepes, pH 7.4, 1.5 M NaCl, 1 mM MgCl_2_, 1 mM EGTA, 1 mM DTT, and 0.05% Triton X-100 and three times with an elution wash buffer containing 50 mM Hepes, pH 7.4, 150 mM NaCl, 1 mM MgCl_2_, 1 mM EGTA, 1 mM DTT, and 0.05% Triton X-100. The proteins were eluted with 40–150 µl elution buffer containing 50 mM Hepes, pH 7.4, 150 mM NaCl, 1 mM MgCl_2_, 1 mM EGTA, 1 mM DTT, 2.5 mM d-Desthiobiotin, and 0.05% Triton X-100. To label SNAP-tagged proteins with SNAP-Surface Alexa Fluor 647 (NEB), 20–40 µM dye was incubated with proteins on beads for 1 h between wash and elution steps. After extensive washing, proteins were eluted in the elution buffer with 300 mM instead of 150 mM NaCl. The concentrations of purified proteins were measured by BSA standard using SDS-PAGE. All purified proteins were snap frozen in liquid nitrogen and stored at −80°C.

For protein purification of MAP7D3 constructs from bacteria, BL21 *E. coli* were transformed with the respective MAP7D3 construct. Bacteria were grown to an OD600 of 0.6 at 37°C, after which protein expression was induced with 1 mM IPTG for 1 h at 37°C and 2.5 h at 20°C. Bacteria were spun down and subjected to one freeze–thaw cycle at −80°C to stimulate proper lysis. Bacteria were resuspended and sonicated at 4°C in cold lysis buffer containing 50 mM sodium phosphate, pH 8, 250 mM NaCl, 1 mM MgCl_2_, 1 mM DTT, 0.5% Triton X-100, 1 mM PMSF, and cOmplete protease inhibitor cocktail (Roche). Lysates were centrifuged at 25,000 *g* for 45–60 min, and the supernatants were incubated with Strep-Tactin Superflow high-capacity beads (IBA Lifesciences) for 60 min at 4°C, followed by five washes with a buffer containing 50 mM sodium phosphate, pH 6.0, 250 mM NaCl, 1 mM MgCl_2_, and 1 mM DTT. The proteins of interest were eluted with a buffer containing 50 mM sodium phosphate, pH 7.0, 250 mM NaCl, 1 mM MgCl_2_, 1 mM DTT, and 5 mM d-Desthiotiotin. The eluted fractions were pooled and supplemented with 10% sucrose for preservation. Proteins were snap frozen and stored at −80°C. The concentrations of purified proteins were measured by BSA standard using SDS-PAGE.

For protein purification of the minimal MAP7, MAP7D3, and KIF5B fragments used for probing complex formation (SEC-MALS and ITC), all protein constructs were fused to an N-terminal thioredoxin-6xHis cleavable tag by restriction free positive selection into a pET-based bacterial expression vector. For expression, the KIF5B and MAP7D3 constructs were transformed using *E. coli* expression strain BL21(DE3), whereas for the MAP7 construct, BL21-CodonPlus (DE3)-RIPL–competent cells were chosen. All transformed cells were cultivated in Lysogeny broth containing 50 µg/ml kanamycin. Subsequently, upon reaching an OD600 of 0.4–0.6, the cultures were induced with 1 mM IPTG (Sigma-Aldrich) after cooling to 20°C for KIF5B and MAP7D3 or at 37°C for MAP7, respectively. The KIF5B and MAP7D3 constructs were expressed at 20°C overnight, whereas the MAP7 construct was expressed for 4 h at 37°C. Cells were harvested by centrifugation at 4,000 *g* at 4°C followed by sonication for lysis in the buffer containing 50 mM Hepes, pH 7.5, 500 mM NaCl, 10 mM imidazole, 10% glycerol, 2 mM β-mercaptoethanol, protease inhibitors (Roche), and DNaseI (Sigma-Aldrich). The lysate was cleared by centrifugation at 18,000 *g* for 20 min and subsequent filtration through a 0.45-µm filter. All constructs were purified by immobilized metal-affinity chromatography on HisTrap HP Ni^2+^ Sepharose columns (GE Healthcare) at 4°C according to the manufacturer’s instructions. The elutions were cleaved by 3C protease during dialysis against the same buffer as above lacking protease inhibitors and DNase I. The cleaved constructs were separated from tag and protease by a reverse immobilized metal-affinity chromatography run followed by a gel filtration run on a HiLoad Superdex 200 16/60 size exclusion chromatography column (GE Healthcare) in a buffer containing 10 mM Tris HCl, pH 7.5, and 150 mM NaCl. Fractions containing the constructs were pooled and concentrated by ultracentrifugation before being flash frozen and stored at −80°C.

For recombinant protein production of MAP7-Ct(mini), BL21(DE3) Rosetta2 *E. coli* cells (Novagen) containing a pET24a vector (Novagen) encoding a SUMO-MAP7-Ct(mini) construct were cultured. 2 liters of culture, in Lysogeny broth medium supplemented with the antibiotics 10 g/liter kanamycin (Sigma-Aldrich) and 33 g/liter chloramphenicol (Sigma-Aldrich), were grown until OD600 of ∼0.8–1, after which protein production was induced with 0.1 mM IPTG (Thermo Fisher Scientific). Protein production was performed overnight at 18°C. Cells were harvested by centrifugation and subjected to one freeze–thaw cycle at −80°C to initiate cell lysis. The pellet was thawed and resuspended in 50 mM sodium phosphate buffer, pH 8.0, 150 mM NaCl, cOmplete protease inhibitor cocktail (Roche) and 5 mM β-mercaptoethanol. The cells were then disrupted by an EmulsiFlex-C5 (Avestin) cell disruptor. The lysate was cleared by centrifugation (55,000 *g*, 45 min), filtered through a 0.22-µm polypropylene filter (VWR) and mixed for 15 min with Protino Ni-IDA resin (Macherey-Nagel) at 4°C. After centrifugation, the protein was eluted with 50 mM sodium phosphate, pH 8.0, 250 mM imidazole, 150 mM NaCl, cOmplete protease inhibitor cocktail (Roche), and 5 mM β-mercaptoethanol. To cleave off the SUMO tag, the eluate was then digested with Ulp1 overnight at 4°C while dialyzed against 50 mM phosphate buffer, pH 8.0, with a 6-kD cutoff membrane (Spectrum Laboratories). The protein was loaded on a POROS 20HS (Thermo Fisher Scientific) cation exchange column in the same dialysis buffer using an ÄKTA Purifier chromatography system (GE Healthcare). The protein was eluted by a linear gradient up to 2 M KCl over 15 CV (Carl Roth); fractions of 0.5 ml were collected. Fractions of interest were then concentrated and exchanged against 25 mM Hepes buffer, pH 7.5, with 75 mM KCl, 75 mM NaCl, and 10 mM DTT using a Vivaspin column (cutoff: 6 kD). The final concentration was determined by an ND-100 spectrophotometer (Nanodrop Technologies). Purity was confirmed by SDS-PAGE, and the protein was aliquoted and stored at −80°C.

### Mass spectrometry

After streptavidin purification, beads were resuspended in 20 µl Laemmli sample buffer (Biorad), and supernatants were loaded on a 4–12% gradient Criterion XT Bis-Tris precast gel (Biorad). The gel was fixed with 40% methanol/10% acetic acid and then stained for 1 h using colloidal Coomassie dye G-250 (Gel Code Blue Stain Reagent; Thermo Fisher Scientific). After in-gel digestion, samples were resuspended in 10% formic acid/5% DMSO and analyzed using an Agilent 1290 Infinity (Agilent Technologies) liquid chromatography, operating in reverse-phase (C18) mode, coupled to an Orbitrap Q-Exactive mass spectrometer (Thermo Fisher Scientific). Peptides were loaded onto a trap column (Reprosil C18, 3 µm, 2 cm × 100 µm; Dr. Maisch) with solvent A (0.1% formic acid in water) at a maximum pressure of 800 bar and chromatographically separated over the analytical column (Zorbax SB-C18, 1.8 µm, 40 cm × 50 µm; Agilent) using a 90-min linear gradient from 7–30% solvent B (0.1% formic acid in acetonitrile) at a flow rate of 150 nl/min. The mass spectrometer was used in a data-dependent mode, which automatically switched between mass spectrometry and tandem mass spectrometry. After a survey scan from 350 to 1,500 m/z, the 10 most abundant peptides were subjected to higher-energy collisional dissociation fragmentation. Mass spectrometry spectra were acquired in high-resolution mode (R > 30,000), whereas MS2 was in high-sensitivity mode (R > 15,000). Raw files were processed using Proteome Discoverer 1.4 (version 1.4.0.288; Thermo Fisher Scientific). The database search was performed using Mascot (version 2.4.; Matrix Science) against a Swiss-Prot database (taxonomy human). Carbamidomethylation of cysteines was set as a fixed modification, and oxidation of methionine was set as a variable modification. Trypsin was specified as enzyme, and up to two missed cleavages were allowed. Data filtering was performed using percolator, resulting in a 1% false discovery rate. Additional filters were search engine rank 1 peptides and ion score >20.

### SEC-MALS experiments

SEC-MALS experiments were conducted in a buffer containing 10 mM Tris HCl, pH 7.5, supplemented with 150 mM NaCl at 20°C using a Superdex 200 10/300 analytical size exclusion chromatography column (GE Healthcare) coupled to a miniDAWN TREOS light scattering and Optilab T-rEX refractive index detector (Wyatt Technology). A volume of 30 µl of protein samples at 5 mg/ml was injected, and data were analyzed with the software provided by the manufacturer.

### ITC

The proteins were dialyzed against a buffer containing 10 mM Tris HCl, pH 7.5, supplemented with 150 mM NaCl overnight at 4°C. ITC experiments were conducted on an iTC200 instrument at 25°C using an injection volume of 2.1 µl at a reference power of 7 and stirring speed of 700 rpm. For probing the MAP7–KIF5B interaction, the MAP7 fragment was used as titrant at a concentration 576 µM against the KIF5B fragment at 15 µM in the cell. The MAP7D3–KIF5B interaction was probed with 300 µM MAP7D3 fragment as titrant and 30 µM KIF5B fragment in the cell. The resulting enthalpograms were integrated and fitted using the standard one-site-model of Origin (OriginLab).

### In vitro reconstitution assays

MT seeds were prepared by incubating 20 µM porcine tubulin mix containing 70% unlabeled, 18% biotin-tubulin, and 12% rhodamine-tubulin with 1 mM GMPCPP at 37°C for 30 min. Polymerized MTs were separated from the mix by centrifugation in an Airfuge at 119,000 *g* for 5 min. MTs were subjected to one round of depolymerization and polymerization in 1 mM GMPCPP, and the final MT seeds were stored in MRB80 buffer (80 mM K-Pipes, pH 6.8, 1 mM EGTA, and 4 mM MgCl_2_) containing 10% glycerol. In vitro reconstitution assays were performed in flow chambers assembled from microscopy slides and plasma-cleaned coverslips. The chambers were treated with 0.2 mg/ml PLL-PEG-biotin (Surface Solutions) in MRB80 buffer for 5 min. After washing with the assay buffer, they were incubated with 1 mg/ml NeutrAvidin for 5 min. MT seeds were attached to the biotin-NeutrAvidin links and incubated with 1 mg/ml κ-casein. The in vitro reaction mixture consisted of 20 µM tubulin, 50 mM KCl, 0.1% methylcellulose, 0.5 mg/ml κ-casein, 1 mM GTP, an oxygen scavenging system (20 mM glucose, 200 µg/ml catalase, 400 µg/ml glucose-oxidase, and 4 mM DTT), 2 mM ATP, 0.2–10 nM of the respective kinesin (concentrations were calculated for monomeric proteins), and MAP7 or MAP7D3 at indicated concentrations. After centrifugation in an Airfuge for 5 min at 119,000 *g*, the reaction mixture was added to the flow chamber containing the MT seeds and sealed with vacuum grease. The experiments were conducted at 30°C, and data were collected using total internal reflection fluorescence (TIRF) microscopy. For some experiments without mCherry-labeled MAP7/MAP7D3, the reaction mixture was composed of 19.5 µM tubulin supplemented with 0.5 µM rhodamine-labeled tubulin. All tubulin products were purchased from Cytoskeleton Inc.

For MT pelleting assays, a reaction containing 37.5 µM porcine brain tubulin supplemented with 1 mM GTP, 1 mM DTT, and 20 µM Taxol in MRB80 was prepared at 30°C for 30 min. The reaction was divided and supplemented with MRB80 buffer with or without mCherry-MAP7-Ct at a final tubulin concentration of 30 µM. Also a control without tubulin was included. Subsequently, all reactions were incubated for another 15 min at 30°C. Pelleting was performed in an Airfuge at 119,000 *g* with a prewarmed rotor for 10 min. Supernatants were removed and pellets were resuspended in MRB80 buffer on ice by regular pipetting for 40 min. All samples were supplemented with 4× Laemmli sample buffer, boiled, and analyzed by SDS-PAGE.

### Image acquisition

Fixed cells were imaged with a Nikon Eclipse 80i upright fluorescence microscope equipped with Plan Apo VC NA 1.40 oil 100× and 60× objectives or a Nikon Eclipse Ni-E upright fluorescence microscope equipped with Plan Apo Lambda 100× NA 1.45 oil and 60× NA 1.40 oil objectives microscopes, Chroma ET-BFP2, -GFP, -mCherry, or -Cy5 filters, and a Photometrics CoolSNAP HQ2 charge-coupled device (CCD) camera (Roper Scientific). The microscopes were controlled by Nikon NIS Br software.

FRAP and LEXY optogenetic experiments were done using spinning disk microscopy, which was performed on an inverted research microscope Eclipse Ti-E with the Perfect Focus System (Nikon), equipped with Plan Apo VC 100× NA 1.40 and Plan Apo 60× NA 1.40 oil objectives, a Yokogawa CSU-X1-A1 confocal head with 405-491-561 triple-band mirror, and GFP, mCherry, and GFP/mCherry emission filters (Chroma), ASI motorized stage MS-2000-XYZ with Piezo Top Plate (ASI), a Photometrics Evolve 512 electron-multiplying CCD camera (Photometrics), and controlled by MetaMorph 7.7 software (Molecular Devices). The microscope was equipped with a custom-ordered illuminator (MEY10021; Nikon) modified by Roper Scientific France/PICT-IBiSA, Institut Curie. Cobolt Calypso 491 nm (100 mW) and Cobolt Jive 561 nm (100 mW) lasers (Cobolt) were used as light sources. To keep cells at 37°C, we used a stage top incubator (model INUBG2E-ZILCS; Tokai Hit).

All in vitro reconstitution assays were imaged on an inverted research microscope Nikon Eclipse Ti-E with the perfect focus system (Nikon) equipped with Nikon CFI Apo TIRF 100× 1.49 NA oil objective, Photometrics Evolve 512 EMCCD (Roper Scientific), and Photometrics CoolSNAP HQ2 CCD (Roper Scientific) and controlled with MetaMorph 7.7 software (Molecular Devices). The microscope was equipped with TIRF-E motorized TIRF illuminator modified by Roper Scientific France/PICT-IBiSA, Institut Curie. For excitation lasers we used a 491-nm 100-mW Stradus (Vortran), 561-nm 100-mW Jive (Cobolt), and 642-nm 110-mW Stradus (Vortran). We used an ET-GFP 49002 filter set (Chroma) for imaging of proteins tagged with GFP, an ET-mCherry 49008 filter set (Chroma) for imaging X-rhodamine–labeled tubulin or mCherry-tagged proteins, and an ET-405/488/561/647 for imaging SNAP–Alexa Fluor 647. For simultaneous imaging of green and red fluorescence, we used an Evolve512 EMCCD camera (Photometrics) and ET-GFP/mCherry filter cube (59022; Chroma) together with an Optosplit III beamsplitter (Cairn Research Ltd) equipped with double-emission filter cube configured with ET525/50m, ET630/75m, and T585lprx (Chroma). For simultaneous imaging of green, red, and far-red fluorescence, we used an Evolve512 EMCCD camera (Photometrics), quad TIRF polychroic ZT405/488/561/640rpc (Chroma), and quad laser emission filter ZET405/488/561/635m (Chroma), mounted in the metal cube (Chroma, 91032), together with an Optosplit III beamsplitter (Cairn Research Ltd) equipped with triple-emission filter cube configured with ET525/50m, ET630/75m, and ET700/75m emission filters and T585lprx and T660lprx dichroic (Chroma). To keep in vitro samples at 30°C, we used a stage top incubator (model INUBG2E-ZILCS).

### Image processing and analysis

Images and movies were processed using ImageJ. All images were modified by linear adjustments of brightness and contrast. Maximum intensity projections were made using z projection. Kinesin velocities, run lengths, and landing frequencies were obtained from kymograph analysis using ImageJ plugin KymoResliceWide v.0.4 (https://github.com/ekatrukha/KymoResliceWide; copy archived at https://github.com/elifesciences-publications/KymoResliceWide). Kinesin runs <0.5 s were included for landing frequency analysis but not analyzed for run length and velocity. Kinesins running on GMPCPP MT seeds were excluded from our analysis as much as possible. Kinesin runs >2.0 s were analyzed for MAP7/MAP7D3 cotransport events.

For quantifying mitochondria in different HeLa cell knockdown/knockout conditions, we classified mitochondria as “clustered” when ∼80% of the cytochrome *c* signal was localized in a dense cluster around the nucleus, whereas all other localization patterns with more spread mitochondria were classified as “spread.” K560-GFP was classified as localized on corner MTs when clear enrichment of fluorescent signal was seen at MT ends near the cell periphery over MTs that are localized in between the boundary and the cell center.

Line-scan analysis of MT localization of MAP7 linker truncations was performed on widefield images of HeLa and COS7 cells overexpressing GFP-tagged constructs costained for α-tubulin. Average fluorescence intensities were measured from 1- to 3-µm line scans along MTs stained for α-tubulin and an adjacent line 5 pixels away from the same MT as a background intensity measurement. Next, the ratio was calculated by dividing fluorescent intensity of the MT line scan by the fluorescent intensity of the control line scan at the same region. No enrichment of the GFP-tagged construct on MTs would result in an intensity ratio of 1. Per condition, 30 MTs were analyzed from 10 cells (3 MTs per cell).

Imaging of MT plus ends with EB3-GFP as a marker was performed on a TIRF microscope. Imaging was performed at two frames per second for 50 s. Per cell, ∼10–30 EB3 comets were traced, and average growth velocity/duration was calculated and analyzed per cell using SigmaPlot.

FRAP measurements were performed by bleaching a 10 × 10-µm-square region in a cytoplasmic region between the nucleus and cell cortex followed by 8.5-min imaging with a frame interval of 3 s. Mean fluorescence intensities were measured from a 4 × 4-µm-square region within the original photobleached region to avoid analyzing nonbleached MTs that could slide into the analyzed region. The mean intensity of this region was double corrected for background fluorescence and photobleaching (Phair et al., 2004).

Optogenetics experiments with blue light–inducible K560-LEXY kinesin were performed using spinning disk microscopy. Prior to imaging, a round of MT disassembly was performed to promote nuclear import of K560-LEXY by treating the cells with 10 µM nocodazole (Sigma-Aldrich) for 1 h at 37°C, followed by 1 h at 4°C, followed by an extensive washout with prewarmed medium to reassemble MTs. Acquisitions were done with a frame interval of 5 s after sequential exposure with a green-light 561-nm laser (to image kinesin) followed by a blue-light 491-nm laser (to image MAPs and activate K560-LEXY simultaneously). Exposure times of ∼1 s per interval with the 491-nm laser were sufficient to induce active export of LEXY-tagged motors from the nucleus. For measuring fluorescence intensity changes at cell corners, a maximum intensity projection of the K560-LEXY channel over time was made using ImageJ, followed by Gaussian blurring and thresholding to select cell corners to analyze. Mean fluorescence values for GFP-MAP7/MAP7D3 and K560-LEXY were obtained from the same cell corners over time, background subtracted, and normalized to the mean fluorescence in that region at t = 0 min. Changes in mean fluorescence intensity were plotted per cell corner.

### Single-molecule intensity analysis

Single-molecule fluorescence histograms of monomeric GFP (control) or kinesins moving on MT lattices were built from acquisitions made on a TIRF microscope. To ensure identical imaging conditions, a single imaging slide (with a plasma cleaned coverslip) was used containing two or three flow chambers to image GFP (control) and K560-GFP (with or without MAP7 proteins; Fig. S4 C) or K370-GFP and K560-GFP (Fig. S5 D). For purified GFP, the protein was diluted in MRB80 and added to an imaging flow chambers; chambers were subsequently washed with MRB80, leaving a fraction of the GFP proteins immobilized on the coverslip. Protein dilution was optimized to provide images of ∼0.01 fluorophores/µm^2^ for GFP control conditions. To estimate the number of GFP molecules per kinesin, an in vitro reconstitution assay with K370-GFP or K560-GFP moving on MTs in the presence or absence of MAP7 proteins was set up in the other flow chambers as described before. After sealing with vacuum grease to prevent evaporation, samples were imaged at 30°C. For monomeric GFP, ∼100 images were acquired at different positions on the coverslip to avoid prebleaching. For moving kinesins, ∼5–10 movies were obtained where only the first 10 frames were analyzed to prevent analyzing partially photobleached motors. All acquisitions were obtained under identical laser power, exposure time, and TIRF angle. ImageJ plugin Comdet v.0.3.6.1 and DoM_Utrecht v.1.1.5 (https://github.com/ekatrukha/DoM_Utrecht) were used for detection and fitting of single-molecule fluorescent spots as described previously (Yau et al., 2014). In short, individual spots were fitted with 2D Gaussian, and the amplitude of the fitted Gaussian function was used as a measure of the fluorescence intensity value of an individual spot. The histograms were fitted to lognormal distributions using GraphPad Prism 7.

### Statistical analysis

Statistical significance was analyzed either using the Mann–Whitney *U* test or *t* test, as indicated in the figure legends. For the *t* test, data distribution was assumed to be normal, but this was not formally tested. Statistical significance was determined using GraphPad Prism software (version 7.04). Fitting of run lengths with the sum of two or three exponential decays was performed on the raw data using maximum likelihood estimation method implemented in *mle* function of MATLAB R2011b (MathWorks).

### Online supplemental material

Fig. S1 shows the analysis of MT organization and dynamics in MAP7-depleted cells, the distribution of MAP7D3-Ct in HeLa cells, uncropped images of the MT pelleting assay from Fig. 2 B, and in vitro assays with mCherry-MAP7-Ct. Fig. S2 illustrates the overexpression of GFP-tagged MAP7 linker constructs in COS7 cells and overexpressed N and C terminus constructs of MAP7, MAP7D1, and MAP7D3 together with K560-GFP in HeLa cells depleted of all three MAP7 proteins. Fig. S3 provides an overview of all purified proteins used in this study, the analysis of kinesin purification contaminants and velocity parameters of full-length KIF5B. Figs. S4 and S5 include additional control conditions for the ITC experiment and illustrate binding of MAP7 proteins to MTs in in vitro reconstitution assays as well as quantifications of kinesin behavior from Figs. 5, 6, 7, 8, and 9. Videos 1 and 2 show HeLa cells transfected with K560-LEXY, GFP-MAP7 (Video 1), or GFP-MAP7D3 (Video 2). Exposure with blue light induces nuclear export of K560-LEXY. Total video length is 4 min.

## Supplementary Material

Supplemental Materials (PDF)

Video 1

Video 2
