# Supplementary material for: MAP7 family proteins regulate kinesin-1 recruitment and activation
Source: J Cell Biol. 2019 Feb 15;218(4):1298–318. doi: 10.1083/jcb.201808065 (PMC6446838; doi:10.1083/jcb.201808065)
Supplement: Supplemental Materials (PDF) [file JCB_201808065_sm.pdf]

Supplemental material

Hooikaas et al., https://doi.org/10.1083/jcb.201808065

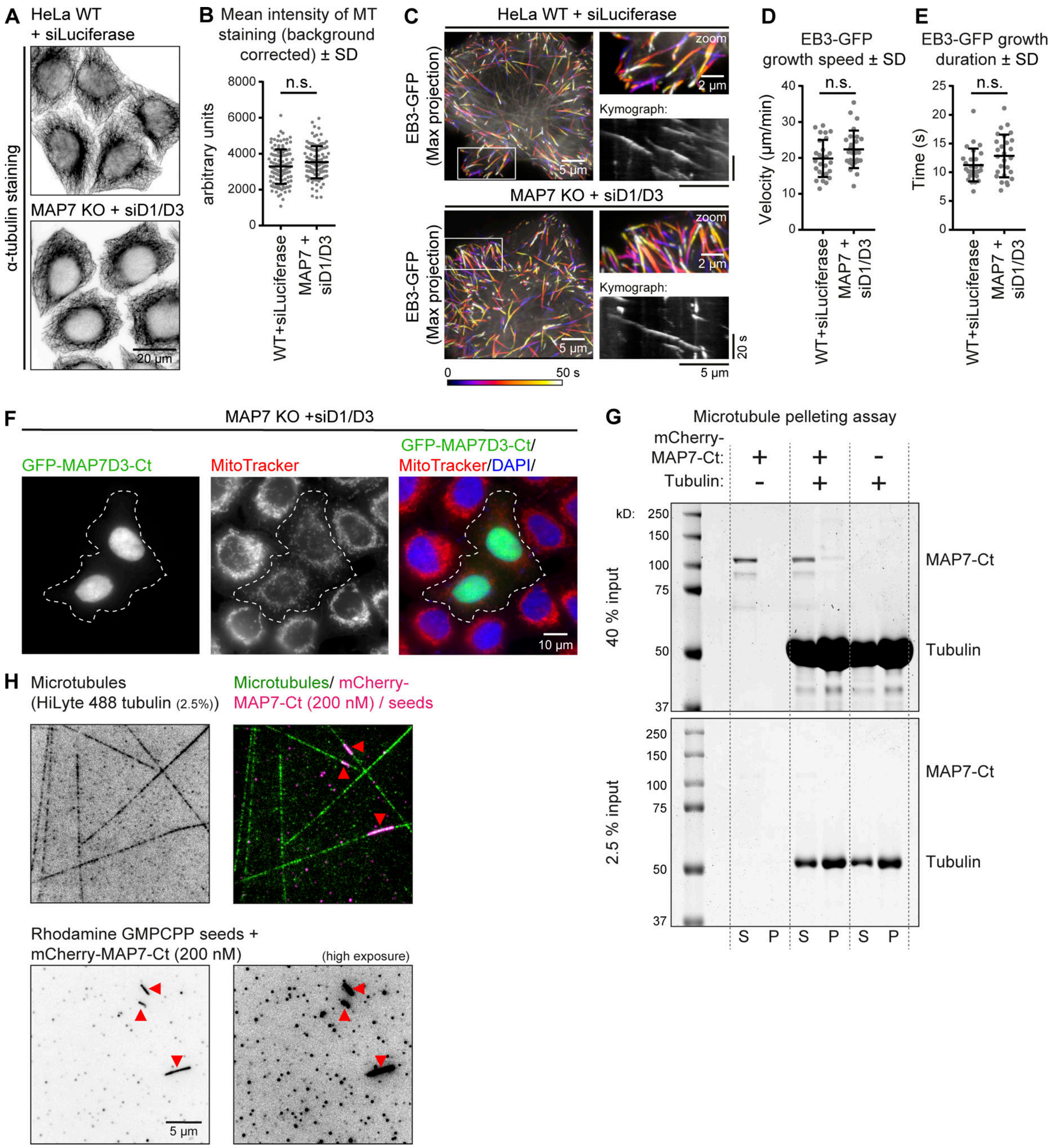

Figure S1. **Effects of the depletion MAP7 family proteins on the MT network and characterization of their MT-binding domains. (A and B)** Control or MAP7 knockout (KO) + siMAP7D1/D3 HeLa cells were stained for α-tubulin and imaged on a widefield microscope (A) to quantify MT intensity per cell (B); *n* = 123 cells (WT + siLuciferase) and *n* = 115 cells (MAP7 KO + siMAP7D1/D3) from three independent experiments, P = 0.054, *t* test. **(C)** Live-cell imaging of EB3-GFP in control or MAP7 KO + siMAP7D1/D3 HeLa cells on a TIRF microscope. Color-coded maximum intensity projections, zooms of the white boxed area, and illustrative kymographs of growing EB3-GFP comets are shown per condition. **(D and E)** Quantification of EB3-GFP dynamics: growth rate (D) and growth duration (E). *n* = 358 comets from 27 cells (WT + siLuciferase) and *n* = 289 comets from 27 cells (MAP7 KO + siMAP7D1/D3) from three independent experiments; (D) P = 0.078 and (E) P = 0.138 (Mann–Whitney *U* test). **(F)** Widefield images of overexpressed GFP-MAP7D3-Ct in MAP7 KO + siMAP7D1/D3 HeLa cells stained with MitoTracker and DAPI to visualize mitochondria and nuclei. **(G)** Unprocessed Coomassie blue–stained SDS-PAGE gel of the MT pelleting assay shown in Fig. 2 B. Two gels were loaded with different input quantities (40% and 2.5% of total samples). The positions of tubulin and mCherry-MAP7-Ct truncation on the gel are indicated on the right. **(H)** Images showing in vitro polymerized MTs, with HiLyte 488–labeled tubulin, rhodamine-labeled MT seeds, and mCherry-MAP7-Ct. Images were obtained on a TIRF microscope. Rhodamine-labeled GMPCPP-stabilized MT seeds are indicated by arrowheads. The mCherry-MAP7-Ct image (also showing MT seeds) is shown on the right with linearly increased brightness/contrast (ImageJ software).

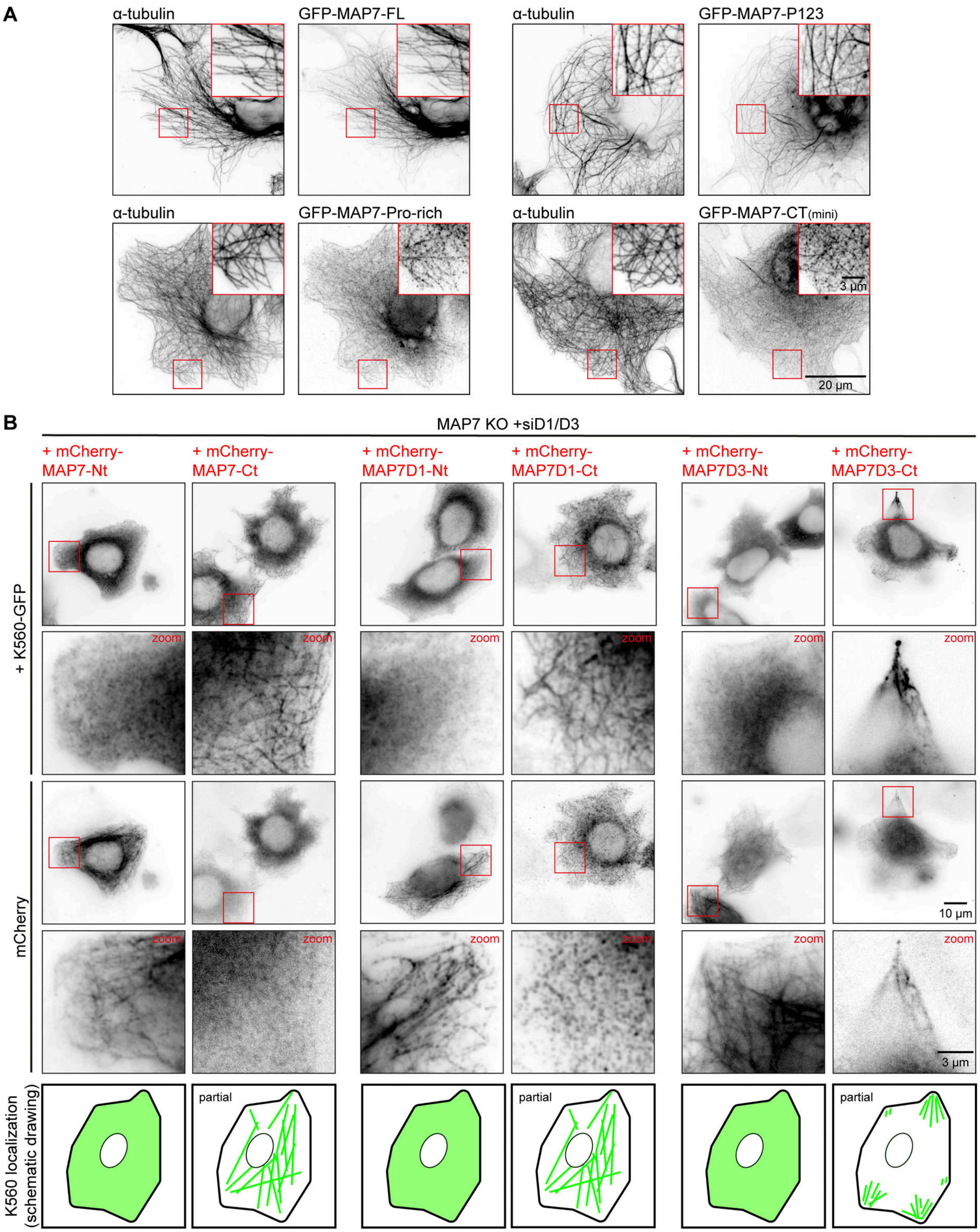

Figure S2. **Kinesin-1 recruitment to MTs by the C termini of MAP7 family proteins. (A)** COS7 cells overexpressing indicated GFP-tagged MAP7 constructs costained for α-tubulin. Zooms are indicated with red boxes. **(B)** Widefield images of MAP7 KO + siMAP7D1/D3 HeLa cells overexpressing K560-GFP with mCherry-tagged MAP7 N- and C-terminal constructs. Enlargements of images indicated with a red squared box are shown in the panel row below. A schematic and representative drawing of K560-GFP localization for each condition is shown at the bottom. KO, knockout.

https://doi.org/10.1083/jcb.201808065

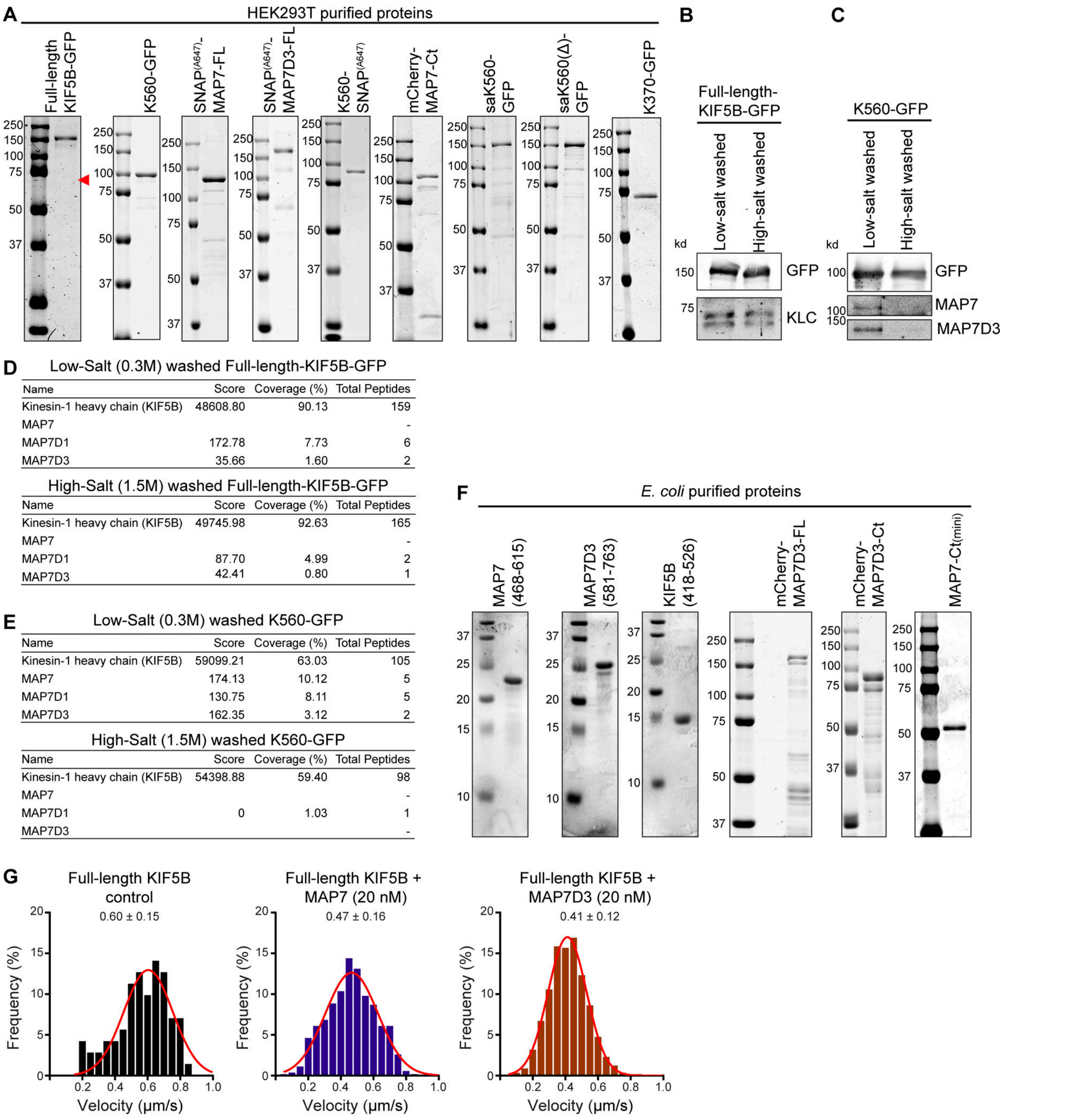

D Low-Salt (0.3M) washed Full-length-KIF5B-GFP

Name	Score	Coverage (%)	Total Peptides
Kinesin-1 heavy chain (KIF5B)	48608.80	90.13	159
MAP7			-
MAP7D1	172.78	7.73	6
MAP7D3	35.66	1.60	2

High-Salt (1.5M) washed Full-length-KIF5B-GFP

Name	Score	Coverage (%)	Total Peptides
Kinesin-1 heavy chain (KIF5B)	49745.98	92.63	165
MAP7			-
MAP7D1	87.70	4.99	2
MAP7D3	42.41	0.80	1

E Low-Salt (0.3M) washed K560-GFP

Name	Score	Coverage (%)	Total Peptides
Kinesin-1 heavy chain (KIF5B)	59099.21	63.03	105
MAP7	174.13	10.12	5
MAP7D1	130.75	8.11	5
MAP7D3	162.35	3.12	2

High-Salt (1.5M) washed K560-GFP

Name	Score	Coverage (%)	Total Peptides
Kinesin-1 heavy chain (KIF5B)	54398.88	59.40	98
MAP7			-
MAP7D1	0	1.03	1
MAP7D3			-

Figure S3. **Overview and analysis of purified proteins. (A)** Proteins purified from HEK293T cells used in this study analyzed by SDS-PAGE. **(B and C)** Western blot analysis of purified kinesins washed with low- (0.3 M) or high-salt (1.5 M NaCl) buffer. Antibodies against GFP, kinesin light chain-1 (KLC), MAP7, and MAP7D3 were used; GFP serves as a loading control for both experiments. **(D and E)** Mass spectrometry analysis of purified kinesins washed with low- (0.3 M) or high-salt (1.5 M NaCl) buffer. **(F)** Proteins purified from *E. coli* used in this study were analyzed by SDS-PAGE. **(G)** Histograms of full-length kinesin-1 velocities in control conditions or in the presence of 20 nM MAP7 or MAP7D3. Red lines show fitting with Gaussian distributions; mean values ± SD are indicated in the plot. n = 71 (control), n = 542 (MAP7), and n = 568 (MAP7D3) from two or three independent experiments.

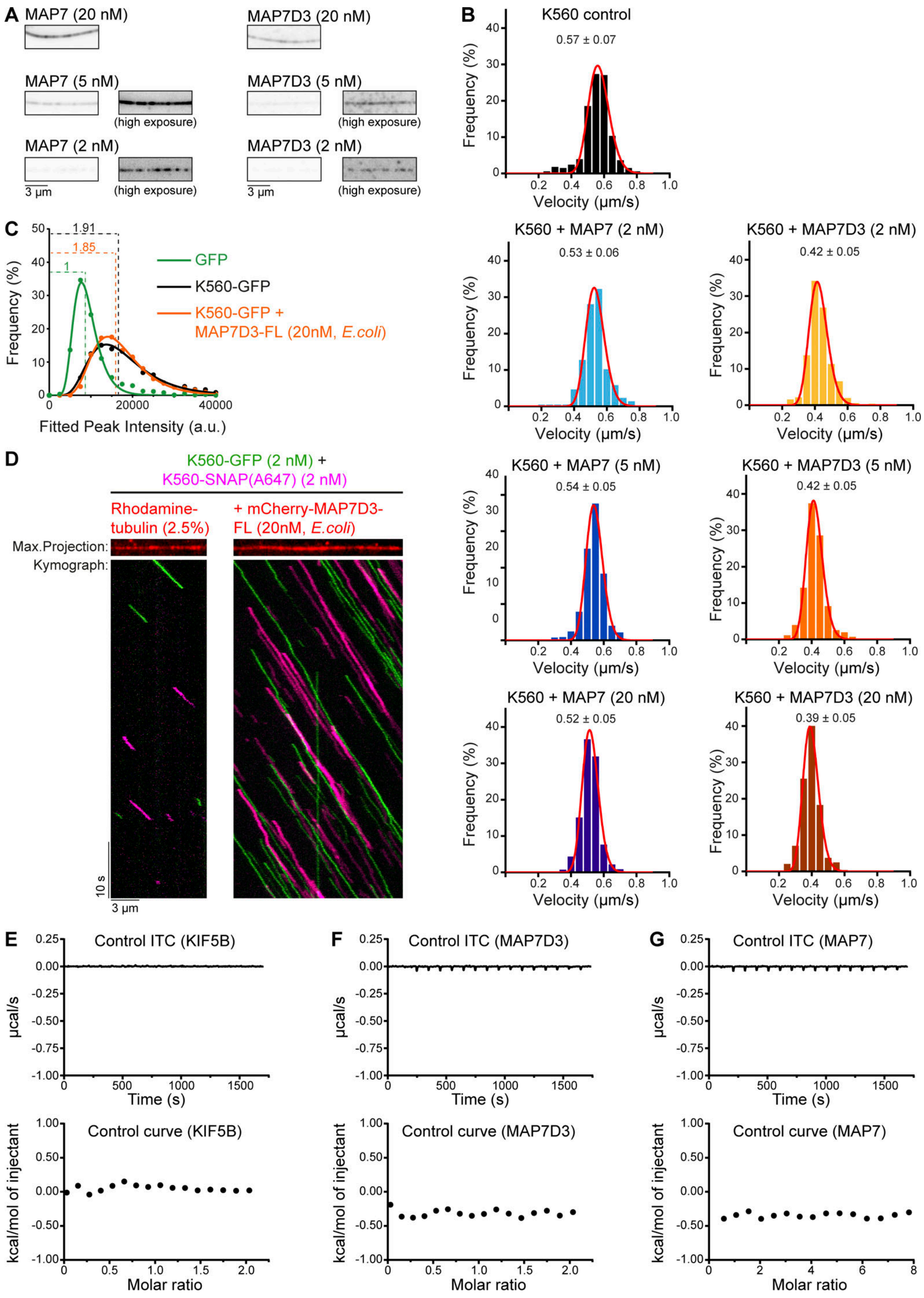

Figure S4. **Velocities and oligomerization state of kinesin constructs in different conditions. (A)** Images showing increasing concentrations of SNAP(Alexa Fluor 647)–tagged MAP7 or MAP7D3 on dynamic MTs in vitro. Images were obtained with identical laser power and exposure time on a TIRF microscope. Panels on the right show replicate images from the left with linearly increased brightness/contrast (ImageJ software). **(B)** Histograms of K560-GFP velocities in control conditions or in the presence of indicated proteins. Red lines show fitting with Gaussian distributions; mean values with standard deviation are indicated in the plot. *n* = 241 (control), *n* = 351 (MAP7, 2 nM), *n* = 614 (MAP7, 5 nM), *n* = 361 (MAP7, 20 nM), *n* = 257 (MAP7D3, 2 nM), *n* = 436 (MAP7D3, 5 nM), and *n* = 303 (MAP7D3, 20 nM) from two independent experiments. **(C)** Histograms of fluorescence intensities of single GFP molecules (immobilized on coverslips) and K560-GFP moving on MTs with or without mCherry-MAP7D3-FL (purified from *E. coli*) in two separate chambers on the same coverslip (dots) and the corresponding fits with lognormal distributions (lines). *n* = 858 (GFP), *n* = 1640 (K560-GFP), and *n* = 4137 molecules (K560-GFP + mCherry-MAP7D3-FL). Fluorophore density was ~0.01 μm^{-2} for GFP. K560-GFP proteins were analyzed from 2 to 10 MTs per movie. Dashed lines show corresponding relative median values. **(D)** Representative kymographs of 1:1 mixed K560-GFP (green) and K560-SNAP(Alexa Fluor 647) (magenta) moving on dynamic MTs with or without mCherry-MAP7D3-FL (purified from *E. coli*). Maximum intensity projections show rhodamine-labeled MTs (control) or mCherry-MAP7D3-FL labeled MTs in red. **(E–G)** Control ITC experiments of KIF5B, MAP7D3, and MAP7. Top: Enthalpograms of the respective titrations. Bottom: Black dots represent the integrated heat change.

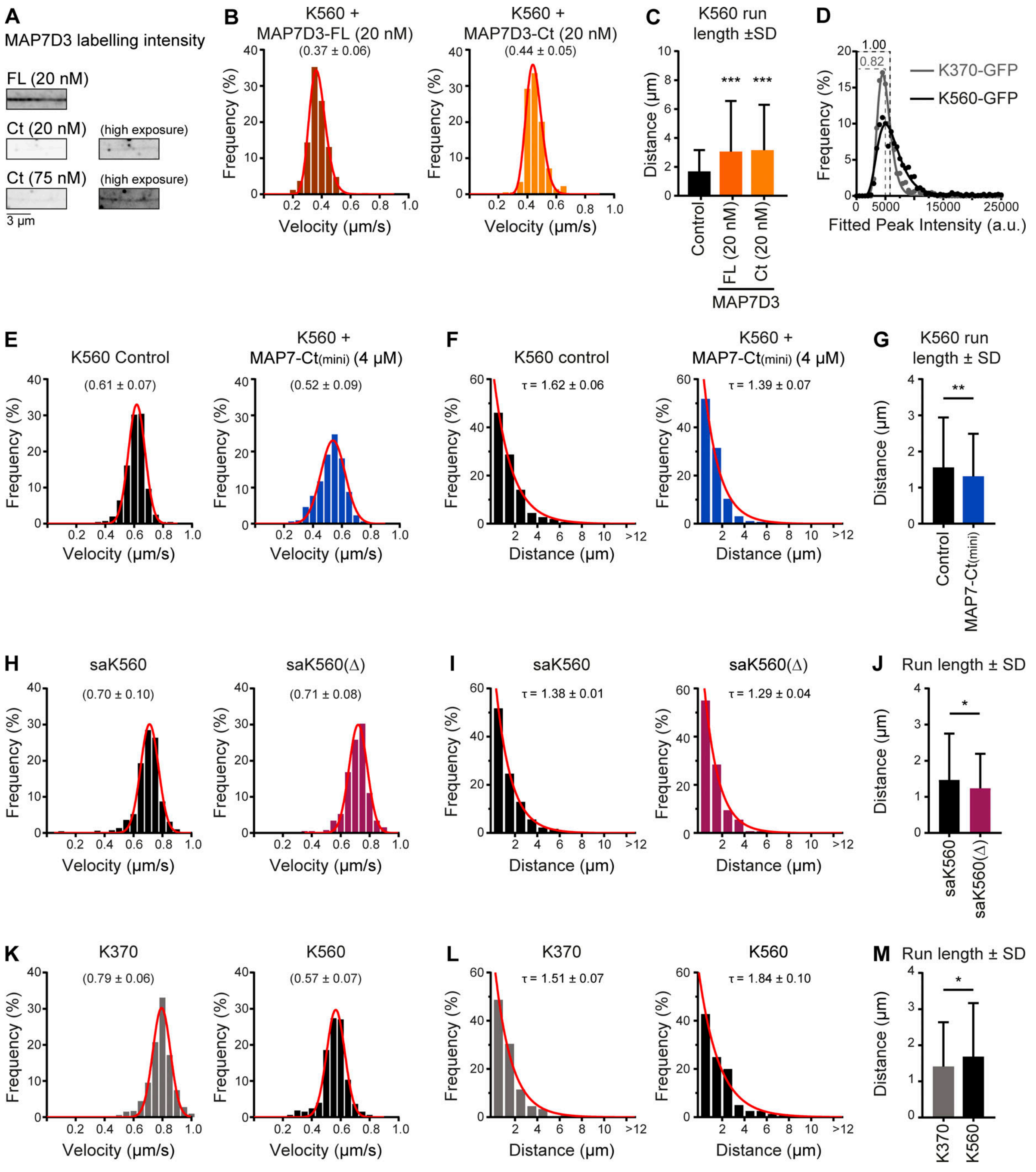

Figure S5. **Kinesin motility parameters. (A)** Images showing increasing concentration of mCherry-tagged MAP7D3 full-length (FL) or MAP7D3-Ct (purified from *E. coli*) on dynamic MTs in vitro. Images were obtained with identical laser power and exposure time on a TIRF microscope. Panels on the right show replicate images from the left with linearly increased brightness/contrast (ImageJ software). **(B)** Histograms of K560-GFP velocities in the presence of indicated MAP7D3 proteins (purified from *E. coli*). Red lines show fitting with Gaussian distributions; mean values with standard deviation are indicated in the plot. n = 271 (MAP7D3-FL) and n = 209 (MAP7D3-Ct). **(C)** Quantification of K560-GFP run length in control condition or in the presence of the indicated MAP7D3 proteins (purified from *E. coli*). *** $P < 0.001$, Mann–Whitney U test. n = 241 (control), n = 271 (MAP7D3-FL, 20 nM), n = 209 (MAP7D3-Ct, 20 nM). **(D)** Histograms of fluorescence intensities of K370-GFP and K560-GFP motors moving on MTs in two separate chambers on the same coverslip (dots) and the corresponding fits with lognormal distributions (lines). n = 639 (K370-GFP) and n = 1,337 molecules (K560-GFP); motor proteins were analyzed from 2–10 MTs per movie. Dashed lines show corresponding relative median values. **(E, H, and K)** Histograms of kinesin velocities. Red lines show fitting with Gaussian distributions; mean values with standard deviation are indicated in the plot. (E) n = 404 (control) and n = 648 (MAP7-Ct(mini)); (H) n = 804 (saK560) and n = 380 (saK560(Δ)); (K) n = 723 (K370) and n = 241 (K560). **(F, G, I, J, L, and M)** Kinesin run lengths were quantified and are shown as a histogram distribution with a fitted exponential decay curve (red), with indicated rate constants (tau) as a measure of mean run length (F, I, and L) or with a bar graph (G, J, and M) **, $P < 0.01$; *, $P < 0.05$, Mann–Whitney U test. Numbers (n) correspond to those of the preceding panels showing kinesin velocities.

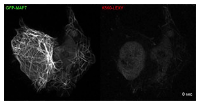

Video 1. **Imaging of light-induced nuclear export of K560-LEXY with GFP-MAP7.** Sequential dual-color video of GFP-MAP7 (left) and K560-mCherry-LEXY (right) in KIF5B KO HeLa cells. The video was acquired at 5 s per frame over the course of 4 min on a spinning disc confocal microscope setup. Video corresponds to Fig. 4 D.

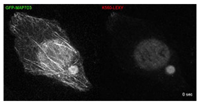

Video 2. **Imaging of light-induced nuclear export of K560-LEXY with GFP-MAP7D3.** Sequential dual-color video of GFP-MAP7D3 (left) and K560-mCherry-LEXY (right) in KIF5B KO HeLa cells. The video was acquired at 5 s per frame over the course of 4 min on a spinning disc confocal microscope setup. Video corresponds to Fig. 4 G.
